# Tackling the outer membrane: facilitating compound entry into Gram-negative bacterial pathogens

**DOI:** 10.1038/s44259-023-00016-1

**Published:** 2023-12-20

**Authors:** Deepanshi Saxena, Rahul Maitra, Rakhi Bormon, Marta Czekanska, Joscha Meiers, Alexander Titz, Sandeep Verma, Sidharth Chopra

**Affiliations:** 1 Department of Molecular Microbiology and Immunology, CSIR-Central Drug Research Institute, Jankipuram Extension, Sitapur Road, Lucknow, 226031 UP India; 2 Department of Chemistry, IIT Kanpur, Kanpur, 208016 UP India; 3 Chemical Biology of Carbohydrates (CBCH), Helmholtz-Institute for Pharmaceutical Research Saarland (HIPS), Helmholtz Centre for Infection Research, 66123 Saarbrücken, Germany; 4 Department of Chemistry, Saarland University, 66123 Saarbrücken, Germany; 5 Deutsches Zentrum für Infektionsforschung (DZIF), 38124 Standort Hannover-Braunschweig, Germany; 6 Center for Nanoscience, IIT Kanpur, Kanpur, 208016 UP India; 7 AcSIR: Academy of Scientific and Innovative Research (AcSIR), Ghaziabad, 201002 India

**Keywords:** Medicinal chemistry, Drug delivery

## Abstract

Emerging resistance to all available antibiotics highlights the need to develop new antibiotics with novel mechanisms of action. Most of the currently used antibiotics target Gram-positive bacteria while Gram-negative bacteria easily bypass the action of most drug molecules because of their unique outer membrane. This additional layer acts as a potent barrier restricting the entry of compounds into the cell. In this scenario, several approaches have been elucidated to increase the accumulation of compounds into Gram-negative bacteria. This review includes a brief description of the physicochemical properties that can aid compounds to enter and accumulate in Gram-negative bacteria and covers different strategies to target or bypass the outer membrane-mediated barrier in Gram-negative bacterial pathogens.

## Introduction

The discovery and clinical utilization of antimicrobials was a very significant turning point in the history of medicine, which significantly reduced morbidity and mortality worldwide. However, this advancement was soon followed by the development of resistance to these antimicrobials, thus raising the prospect of returning to the “pre-antibiotic era” with high lethality associated even with simple infections. This resistance amongst pathogenic microbes is a complex and dynamic process resulting from certain direct or indirect factors including overuse and misuse of antibiotics, poor sanitation, antibiotic disposal, and others. Although antimicrobial resistance (AMR) is a global problem, densely populated areas such as Asia-Pacific regions are at higher risk of spread owing to socioeconomic factors. This increasing threat of multidrug-resistant (MDR) pathogens is widely acknowledged by global and national organizations, including the World Health Organization (WHO) and the Centers for Disease Control and Prevention, USA^1^.

In order to spur antibiotic discovery, WHO has classified carbapenem-resistant Enterobacterales, carbapenem- and colistin-resistant *Acinetobacter baumannii*, and carbapenem-resistant *Pseudomonas aeruginosa* as pathogens of global critical threat in its pathogen priority list. Additionally, the emergence of extensively drug-resistant (XDR) and pan-drug-resistant (PDR) strains of *Klebsiella pneumoniae* worsens the situation^1^. Ameta-analysis of 175 studies conducted in different countries of Southeast Asia revealed 73.0%, 29.8%, and 2.8% cases of carbapenem-resistant *A. baumannii* (CRAB), carbapenem-resistant *P. aeruginosa* (CRPA) and carbapenem-resistant Enterobacterales (CRE), respectively^1^. A survey conducted in 2019 involving 204 countries and territories reported bacterial AMR to be associated with 1.27 million deaths, with the highest death rate recorded in western sub-Saharan Africa (27.3 deaths per 100,000 individuals) and the lowest in Australasia (6.5 deaths per 100,000 individuals). The research highlights the fatality associated with quickly emerging drug-resistant strains of *Escherichia coli*, followed by *Staphylococcus aureus*, *K. pneumoniae*, *Streptococcus pneumoniae*, *A. baumannii*, and *P. aeruginosa*, which were directly responsible for a total of 929,000 deaths^2^. According to a report published by the UK government, AMR will be associated with around 300 million premature deaths, with an associated global economic loss of $100 trillion by 2050^3^. The increasing numbers of MDR bacterial strains, especially Gram-negative bacterial (GNB) pathogens, at an alarming rate highlights the urgent need to develop new antibacterial agents with novel modes of action targeting these pathogens in order to prevent future catastrophic pandemic outbreaks^4^.

Gram-negatives are far more difficult to treat as compared to Gram-positive bacterial (GPB) pathogens because of their uniquely designed lipopolysaccharide (LPS) containing additional outer membrane (OM), their efflux pumps and other resistance mechanisms. Using one or the other mechanism, these bacteria easily bypass the action of most of the commonly used antibiotics and thus, are responsible for a variety of serious community and healthcare-associated infections^5,6^. In this context, the Infectious Diseases Society of America issued a report entitled “Bad Bugs, No Drugs: As Antibiotic Discovery Stagnates, A Public Health Crisis Brews” already in 2004 to encourage and promote pharmaceutical investment in antibiotic research and development^4^. This is however still lagging, with most big pharma companies having left the development of antibiotics and the situation, therefore worsens critically^4,7^.

## The outer membrane of Gram-negative bacterial pathogens as major resistance determinant

### Structural features of outer membrane

The OM of Gram-negative bacteria has a very complex architecture with phospholipids, lipopolysaccharides, lipoproteins and β-barrel porins, combining together to act as an asymmetrical lipid bilayer barrier that prevents the entry of foreign molecules including host defense molecules and antibiotics^8^. The inner leaflet of the OM is made up of phospholipids while the outer leaflet consists of a well-organized, densely packed LPS whose phosphates are stabilized by Mg^2+^/Ca^2+^ ions. LPS consists of the glucosamine-based glycolipid Lipid A and the tight packing of saturated fatty acid chains maintains a low level of membrane fluidity, thus limiting the permeation of hydrophobic compounds across the OM^9^. Additional core and O-antigen oligosaccharides present in LPS of wild-type bacterial strains are required for microbial virulence as they protect bacteria from antibiotics and complement-mediated lysis^10^. Lipoproteins localized on the periplasmic face of OM are involved in various cellular processes such as the transportation of molecules, maintenance of envelope integrity, transduction of signals and pathogenesis^11^. The OM contains certain β-barrel protein channels called porins that act as size exclusion channels, allowing diffusion of small hydrophilic molecules such as nutrients from the extracellular environment into the periplasm but preventing entry of large and/or hydrophobic molecules, including several classes of antibiotics^9^.

### Efflux pumps as major resistance determinant

Unfortunately, even if lipophilic or amphiphilic antibiotics succeed in crossing the OM, there are further lines of defense, e.g., effluxpumps (EPs) that transport these molecules back to the outer environment. These efflux pumps are abundantly used by MDR bacteria to prevent the accumulation of antibiotics in the intracellular environment, thus further decreasing the susceptibility to antibiotics. Naturally, bacteria utilize these pumps to excrete bacterial metabolic products and to recognize harmful exogenous compounds that have penetrated through the cell wall^12^. Efflux pumps were first identified as a resistance mechanism in 1970s^13^ and since then, they are recognized as one of the major factors responsible for, both, intrinsic and acquired resistance in MDR Gram-negative bacterial pathogens. They generally fall under six families based on their mode of activation/regulation and function: 1. ATP-binding cassette (ABC) family, 2. Resistance-nodulation-cell division (RND) family, 3. Major facilitator superfamily, 4. Small multi-drug resistance family, 5. Multi-drug and toxic compound extrusion family and 6. Related proteobacterial antimicrobial compound efflux^14^. All those families of efflux pumps are present in both Gram-positive and Gram-negative bacteria, except for the RND superfamily that is specifically present in Gram-negative bacterial pathogens only and extrude xenobiotics such as antibiotics, dyes, heavy metals, detergents, and many other substrates^12^. Altogether, these unique features of the OM of Gram-negatives combined with the presence of efflux pumps provide a strong defense against commonly used antibiotics^9^.

### Bacterial enzymes targeting antibacterial agents

Apart from these features of the OM and efflux pumps as mechanisms of limited permeability and resistance respectively, Gram-negative bacteria further produce antibiotic modifying enzymes to render them ineffective, such as β-lactamasese.g., serine β-lactamases, metallo-β-lactamases (MBLs) (including Verona integron-encoded metallo-β-lactamases (VIM encoded MBL) and New Delhi metallo-β-lactamases (NDM)), cefotaximase, *K. pneumoniae* carbapenemase (KPC), imipenemase (IMP)and oxacillinase (OXA) that breaks the β-lactam ring. To worsen the situation, bacterial strains producing extended-spectrum β-lactamases (ESBLs) have evolved to target even third-generation cephalosporins, thus further reducing the treatment options available^1^. Besides the β-lactams, also other classes of antibiotics are not left untouched by bacterial enzymatic action. Bacterial ribosome-targeting antibiotics such as aminoglycosides, macrolides, lincosamides and streptogramins are inactivated by bacterial rRNA methyltransferases that modify the drug target site by methylation. The efficacy of peptidoglycan targeting glycopeptides is reduced by peptidoglycan-modifying enzymes such as dehydrogenase, serine racemase, ligase and others. Further worsening the situation, even the last resort antibiotic, colistin is made ineffective by bacterial phosphoethanolamine transferases that modify its target lipid A^15^. Apart from these, thousands of other bacterial enzymes targeting major classes of antibiotics include phosphotransferases, glycosyltransferases and esterases (targeting macrolides), nucleotidyltransferases (targeting lincosamides and aminoglycosides), acetyltransferases (targeting Streptogramin A, phenicols, aminoglycosides and fluoroquinolones), hydrolases (targeting phenicols), phosphotransferases (targeting aminoglycosides, phosphomycin, rifamycins) and monoxygenases (targeting tetracyclins, rifamycins)^16^. A detailedoverviewofthe molecular mechanismofantibioticresistanceamong Gram-negatives has recentlybeenreviewed^17^.

### Membranevesicles, the un-intentionalresistancemechanism

Additionally, both Gram-positive and Gram-negative bacteria possess an additional mechanism of protection through the formation of membrane vesicles (MVs) which are spherical nanoparticles formed of a lipid bilayer. In Gram-negatives, these vesicles are formed by blebbing of OM as a result of cell envelope disruption. While pinching off from the membrane, these vesicles encapsulate some of the periplasmic components of the cell including misfolded proteins, toxic intermediates or foreign substances including certain antibiotics and thus, helping bacteria in detoxification and increasing chances of survival. Sometimes, these vesicles also carry certain antibiotic-inactivating enzymes. However, the molecular mechanism of how these vesicles target antibiotics remains elusive^18^.

Along with OM, inner membrane in Gram-negative bacteria also acts as a permeability barrier for polar and highly charged compounds compared to neutral lipophilic solutes. This phospholipid bilayer also carries a number of solute-specific energy-dependent transporters which restricts the entry of compounds into the cytoplasm. The entry of compounds via inner membrane has been reviewed and is out of scope of this review^19,20^.

### Possible ways of entry of antibiotics in Gram-negative bacteria

Even though a large number of potential intracellular targets have been studied in Gram-negative bacterial pathogens such as peptide deformylase (PDF), enoyl-acyl carrier protein reductase (FabI), 3-ketoacyl-acyl carrier protein III (FabH), methionyl, tRNA synthetase (MetRS) and phenylalanyl-tRNA synthetase (PheRS), identifying inhibitors that accumulate into Gram-negative bacteria has been a major challenge, which is best exemplified by >70 high throughput screening (HTS) campaigns run by GlaxoSmithKline with poor success. This study therefore highlights the limited activity of intracellular-enzyme targeting compounds against Gram-negative bacteria, mainly owing to the extremely limited permeability of OM^21^.

The major routes of entry of compounds into Gram-negative bacteria pathogens are discussed below:

#### Self-promoted uptake

The hydrophobic, fatty acid chain bearing lipid A, a core oligosaccharide and the O-antigen together constitute LPS. The phosphorylated glucosamine backbone of lipid A and the core region carry several anionic groups that strongly attract divalent cations to recompense electrostatic repulsion between neighboring LPS molecules. The mechanism of action of antibacterial agents via self-promoted uptake has already been reviewed^6,22^. Hydrophobic antibacterial agents like macrolides (erythromycin, clarithromycin), rifamycins, novobiocin and fusidic acid make use of the hydrophobic nature of LPS lipid A to enter the bacterial cells. On the other hand, membrane permeabilizers, such as Tris/EDTA, polymyxin B, polymyxin B nonapeptide (PMBN), aminoglycosides (gentamicin, kanamycin) and cationic peptides compete with divalent cations to bind with LPS, resulting in release of LPS into the medium. This reduced LPS in the OM is compensated by glycerophospholipids, which results in phospholipid bilayer patches. These patches are several times more permeable to lipophilic antibiotics. In this way, membrane permeabilizers increase susceptibility of Gram-negative bacteria towards hydrophobic antibiotics^6^.

### Entry via porins

The most common way of entry into Gram-negative bacteria is via hydrophilic β-barrel protein pores. Via these so-called porins, small hydrophilic ions and molecules with a molecular mass below roughly 600 Da, such as amino acids and sugars can diffuse to the bacterial periplasm. The central hydrophilic portion of these proteins makes them compound-selective for hydrophilic molecules. Interestingly, antibiotics like β-lactams, tetracyclines, chloramphenicol and fluoroquinolones find their way into the cell via these porins. However, entry through these porins is affected by physicochemical parameters, e.g., nature and position of specific charges. This dependency was illustrated by Bezrukov’s group, where a sharp increase in the selectivity of the porin OmpF for cations relative to anions was observed in solutions of low ionic strength^6,23^. Interestingly, ampicillin and amoxicillin (both β-lactams) have pH-dependent actions on OmpF porins as they transiently block the open channels of the porins and show maximum blockage at a pH corresponding to their isoelectric point. The interaction between the specific charges of the zwitterionic molecules and the OmpF porins aids the antibiotic drug translocation across the membrane. Quinolone derivatives are bactericidal antibiotics that primarily translocate over the OM via porins (in particular OmpF). Less hydrophobic quinolones like norfloxacin and ciprofloxacin are proven to cross the membrane via other porins^24,25^.

Despite being an excellent way of entry of drugs, studies have revealed the emergence of a large number of resistant strains of *E. coli*, *P. aeruginosa*, *N. gonorrhoeae*, *Enterobacteraerogenes* and *K. pneumoniae* in which either there is loss/severe reduction of porins or replacement of one or two major porins by another type; or altered function due to specific mutations^6^. A wide spread of porin-based drug resistance was reported by Pagès et al. where 44% among 45 β-lactam resistant clinical isolates of *E. aerogenes* obtained from French hospitals showed a lack of porins^26^.

### Facilitated diffusion

Facilitated diffusion is a process of transportation of molecules across the OM utilizing substrate-specific carrier proteins against concentration gradients. The process is independent of metabolic energy but is facilitated by factors like pH and drug concentration. Two such carrier proteins studied in *E. coli* are TonA and TonB. These proteins are naturally used by *E. coli* for the accumulation of ferrichrome (a naturally occurring iron chelator). The antibiotic albomycin, a structural analog of ferrichrome, follows the same path to enter bacterial cell via binding to TonA and TonB^27^.

#### Entry via transport proteins [specific and non-specific proteins]

The OM of Gram-negative bacteria comprises a large number of proteins such as murein lipoprotein (Lpp) (provide OM-peptidoglycan interactions and to maintain OM integrity), OmpA (to maintain the OM integrity) and general diffusion porins. Apart from these proteins, some other specialized protein channels and receptors are found on OM, which are responsible for the uptake of specific substrates. For example, LamB and BtuB help in transportation of maltodextrins and vitamin B12. Proteins such as Omp85 are involved in OM and surface appendage biogenesis. A variety of translocators are employed for the assembly of adhesins, pili and flagella. Additionally, several translocons present in type II secretion systems are involved in the release of toxins and different types of enzymes and proteins are also involved in the assembly of LPS. These transport proteins can be attractive targets for transporting antibiotics. However, most of these targets are not extensively studied as antibiotic entry routes^6^. One exception is siderophore transporters where the first drug, Fetroja (cefiderocol), exploiting this mechanism, combined with facilitated diffusion, for uptake has reached approval. The compound binds to the ferric ion and is transported to the periplasmic space via siderophores^28^.

#### Entry due to Donnan potential

Donnan potential was initially identified as a surface potential in Gram-negative bacteria that are involved in cell mobility and adaptation to different environmental stimuli including charge, ionic strength and pH. Donnan potential is basically the imbalance of charge caused by freely entering, small, permeable ions in presence of large impermeable charged molecules that are trapped in the periplasmic space of Gram-negative bacteria. Generally, membrane potential is negative towards the periplasmic side (40–80 mV), which drives small positive molecules across the membrane. Thus, small positively charged antibacterial compounds can utilize this mode of entry into Gram-negative bacteria^14^.

### Treatment options against Gram-negative bacterial pathogens

Several classes of antibiotics have been developed to target multi-drug resistant Gram-negative bacterial pathogens. For example, fosfomycin was earlier used as an effective treatment option in urinary tract infections (UTIs)^29,30^. Similarly, tigecycline (targeting CRE and CRAB)^31,32^, fluoroquinolones (broad spectrum antibiotic effective even against drug-resistant strains), aminoglycosides (to treat Gram-negative bacteria associated nosocomial pneumonia) and piperacillin/tazobactam (against multiple Gram-negative pathogens)^33^ are other effectively used antibiotics. The membrane-disrupting drug colistin is employed only as an antibiotic of last resort against serious, MDR- Gram-negative bacterial nosocomial infections. However, colistin is highly nephrotoxic and the emergence of colistin-resistant strains limits its clinical use^1^. Tables 1, 2 list various antibiotics discovered or under development targeting Gram-negative bacterial pathogens.

**Table 1:** List of clinically utilized antibiotics to treat infections caused due to Gram-negative bacterial pathogens (Adapted from ref. ^1^).

Standard of care antibiotics		Novel Treatment Options	
Antibiotics	Usage	Antibiotics	Usage
Fosfomycin	UTI^30^	Ceftazidime/avibactam	For HAP & VAP^125^
Tigecycline	CRE and CRAB^126^	Meropenem/vaborbactam	For treating adult cUTIs, including pyelonephritis, cIAIs, and HAP, including VAP^127^
Amikacin, Gentamicin	For GNB-associated nosocomial pneumonia^128^	Ceftolozane/tazobactam	For cIAIs, acute pyelonephritis, cUTIs and HAP, VAP^129^
Piperacillin/tazobactam	Against multiple GNB pathogens^130^	Imipenem/Relebactam	Against the KPC-producing CREs, *K. pneumoniae and P. aeruginosa*^131^
Colistin	MDR bacteria^132^	Cefoperazone/sulbactam	Against Enterobacterales^133^
Levofloxacin, Ciprofloxacin	UTI and others Pharmacotherapy^134^	Cefiderocol	Against a variety of Ambler class A, C and D β-lactamases^135^
		Ertapenem	For treating IAI, cSSSI, cUTI, acute pelvic infections and CAP, limited activity against *P. aeruginosa, A. baumannii* and *B. cepacia n*^136,137^
		Doripenem	Active against AmpC and other ESBL-producing Enterobacteriaceae^138,139^
		Biapenem	Broad spectrum antibiotic targeting *S. pneumoniae*, MSSA, *A. baumannii*, ESBL -producing Enterobacteriaceae, *E. cloacae, S. marcescens* and & *C. freundii*^140^
		Delafloxacin^42^, Lascufloxacin^141,142^	Fluoroquinolone antibiotic approved for the treatment of community-aquired pneumoniaCAP
		Lefamulin	Pleuromutilin derivative for the treatment of community-acquired pneumoniaCAP^143^
		Plazomicin	Aminoglycoside antibiotic approved for the treatment of cUTI^144^
		Relebactam + Imipenem + Cilastatin (Recarbrio)	A DBO-BLI + β-lactam (carbapenem)/degradation inhibitor) for treating cUTI^131^
		Eravacycline	A tetracycline class antibiotic approved for the treatment of IAI^29,145^

*HAP* Hospital-acquired pneumonia, *VAP* Ventilator-associated pneumonia, *UTI* Urinary tract infection, *IAI* intra-abdominal infections, *MBL* metallo-β-lactamase, *cIAI* complicated intra-abdominal infection, *cUTI* complicated urinary tract infection, *cSSSI* complicated skin and skin-structure infection, *CAP* community-acquired pneumonia.

**Table 2:** Description of new molecules being developed against Gram-negative bacterial pathogens^146,147^.

Antibacterial Agents	Lead Compounds	Activity	Developmental stage and Ref
β-lactams			
Imidazole-substituted 6-methylidene-penems	BLI-489	Imparts potent activity against bacterial strains expressing class A and C β-lactamases (AmpC)	Pre-clinical^148^
2-β-alkenyl penam sulfones	48–1220	Potentially targets most common types of β-lactamases	Pre-clinical^149^
4-phenyl cyclic phosphate (β-lactam surrogate)	-	Active against class A and C β-lactamases	Pre-clinical^150^
C3-modified penicillin sulfones	-	Targets class C β-lactamases	Pre-clinical^151^
Monobactam-based compounds	BAL 30376	Displays antibacterial properties against carbapenem-resistant Gram-negative bacteria	Pre-clinical^152^
Tricyclic carbapenem	LK-157	Potentiates activity of aztreonam, ceftazidime and cefuroxime against a wide range of β-lactamase producing Enterobacterales.	Pre-clinical^153^
Oxapenems	AM-112 - AM-115	Active against β-lactamase class A, C and D	Pre-clinical^154^
Diazabicyclooctane (DBO) β-lactamase inhibitor (DBO-BLI)	ARX-1796	Activity against Enterobacterales	Completed Pre-clinical studies^155^
Cephalosporin + DBO-BLI/PBP2 binder combination	Cefepime + Zidebactam	Approved for the treatment of carbapenem resistant gram-negative bacteria including *A. baumannii, P. aeruginosa* and Enterobacterales	Phase III Clinical Trial^156^
DBO-BLI/PBP2 binder + β-lactam-BLI/PBP 1,3 binder	Durlobactam (ETX-2514) + sulbactam	Activity against CRAB	Phase III clinical trial (ATTACK)^157^
Boronate BLI + β-lactam (cephalosporin)	Taniborbactam (VNRX-5133) + cefepime	Activity against CRPA and CRE	Completed Phase III^158^
DBO-BLI/PBP2 binder + β-lactam (carbapenem)	Nacubactam (OP0595) + meropenem	Activity against CRE	Completed Phase I^159^
DBO-BLI/PBP2 binder + β-lactam cephalosporin)	ETX0282 + cefpodoxime	Activity against CRE	Pre clinical^160^
Boronate BLI + β-lactam (cephalosporin)	VNRX-7145 + ceftibuten	Activity against CRE	Completed Phase I^161^
Boronate-BLI + β-lactam (undisclosed)	QPX7728k + QPX2014/QPX7728 + QPX2015	Activity against CRAB, CRE and CRPA	Completed Phase I^162^
NXL104	NXL104/ceftazidime combination	Inactivates β-lactamases via covalent carbamoyl linkage, and is currently under clinical trials for cIAI and cUTI	Completed Phase I^163^
Maleic acid derivates	ME1071 (CP3242)	MBL inhibitor competitively inhibits IMP-1 and VIM-2 producing *E. coli, S. marcescens, A. baumannii* and *K. pneumoniae*	Pre clinical Study^164^
Polymyxins			
	SPR-206	Activity against CRAB, CRPA and CRE	Completed Phase I^165^
	MRX-8	Activity against CRAB, CRPA and CRE	Phase I study^166^
Tetracycline			
	KBP-7072	Activity against CRAB	Phase II study^167,168^
	Amidochelocardin (2-carboxamido-2-deacetyl-chelocardin, CDCHD)	Activity against cUTI-associated ESBL-producing *Enterobacteriaceae*	Pre-clinical study^4,169^
Aminoglycoside			
	EBL-1003 (apramycin)	Activity against CRAB and CRE	Completed Phase I study^170^

*MBL* metallo-β-lactamase, *cIAI* complicated intra-abdominal, *cUTI* complicated urinary tract, *cSSSI* complicated skin and skin-structure infections, *CAP* community-acquired pneumonia, *CRAB* Carbepenem resistant *A. baumannii*, CRE Carbepenem resistant Enterobacterales, CRPA Carbepenem resistant *P. aeruginosa*.

More recently, new antibacterial molecules targeting Gram-negative bacterial pathogens have entered the clinical pipeline. ARX-1796, a diazabicyclooctane (DBO) β-lactamase inhibitor (DBO-BLI) is under development by Arixa Pharmaceuticals for the treatment of Gram-negative *Enterobacterales* infections. Cefepime/Zidebactam is another cephalosporin with a DBO-BLI/PBP2 binder combination approved for treatment of Gram-negative pathogens including *A. baumannii, P. aeruginosa* and *Enterobacterales*^34^.

Apart from these, antimicrobials targeting novel bacterial targets such as bacterial riboswitches (e.g., roseoflovin), quorum sensing (e.g., Gram-negative-specific *N*-acylhomoserine lactones, AHLs), type III secretion systems (e.g., salicylideneacylhydrazides), biofilm (e.g., glycomimetic compounds)^35^, ClpP, ClpX, MgrA, and ToxT modulators (e.g., β-lactone U1, phenyl esters AV170, and AV286) are also under study^36^.

Although numerous classes of antibiotics are available to combat Gram-negative pathogens, rapidly emerging MDR strains limit our treatment options. In this scenario, clinicians are forced to use last-resort drugs such as colistin, etc., consequently increasing the pressure on even last-resort antibiotics^37^.

### Physicochemical parameters of compounds to target outer membrane for accumulation in Gram-negative pathogens

An important step of designing a drug against Gram-negative bacteria is focusing on the increased accumulation of the compound into the bacterial cell. This can be done by tuning the physicochemical parameters of the compound. Several studies have been conducted to study the correlation between the physicochemical properties of compounds and their accumulation in Gram-negatives. Studies involving entry of compounds via porins revealed increased penetration of zwitterionic molecules and reduced penetration of analogs with dianionic charges and bulky/protruding side chains. Thus, highlighting the importance of charge and size of a compound to allow its passage through the constriction zone of porins. Adding on to the physicochemical properties, intracellular accumulation of tetracyclines and fluoroquinolones in *E. coli* was found to display moderate inverse correlation with hydrophobicity^38^.

The initial correlation between the physicochemical properties of a drug and its oral bioavailability was proposed by Lipinski et al. They set five general rules as drugability guidelines for New Molecular Entities (NMEs) based on HTS results. According to these rules, bioavailability of a drug molecule depends on key physico-chemical properties like molecular weight, lipophilicity and hydrogen bond counts^39^. The molecule should possess not more than five hydrogen bond donors, hydrogen bond acceptors (≤10), molecular mass (<500 Da) and calculated octanol-water partition coefficient (clogP ≤ 5). Although the rule is applicable to several drugs, many antibiotics, anti-fungal, vitamins and cardiac glycosides fall outside the “rule of 5”. O’Shea et al. analyzed a set of 147 active antibacterials and reported that anti-bacterial compounds share a different property space as compared to other therapeutic drug classes, i.e., comparatively high molecular weight and low lipophilicity. Narrowing down the antibacterial properties, they pointed out that compounds targeting Gram-positive and Gram-negative bacteria have different physicochemical properties. For instance, the average molecular weight of compounds active against Gram-positive bacteria was 813 Da, while for Gram-negative bacteria, it was 414 Da. Additionally, drugs targeting Gram-negatives were found to have higher polarities (clogP = −0.1) as compared to those targeting Gram-positive bacteria (clogP = 2.1). Higher polarity of compounds targeting Gram-negative bacteria corresponds to the fact that the compounds are seeking their way through the hydrophilic porin channels. Therefore, increased polarity and reduced size aid the entry of a compound into Gram-negative bacteria^40^.

Optimizing the physicochemical parameters of a compound is one of the most challenging ways of initiating the entry of compounds into Gram-negative bacteria. Although low molecular weight and low lipophilicity were identified as two important parameters to potentiate drug entry into Gram-negative bacteria OM via porins, these parameters were not sufficient to develop a broad-spectrum antibacterial drug. Moreover, differences in the LPS composition and OM proteins amongst various bacterial pathogens call for a more exhaustive study^11^.

In this scenario, Hergenrother and colleagues investigated the accumulation of over 180 diverse compounds, including ~70 Gram-positive-only antibiotics in *E. coli* MG1655 to identify the common physicochemical properties of compounds to get accumulated within cells. Based on their observation, they proposed general rules of compound accumulation for *E. coli*, i.e., the presence of an ionizable Nitrogen, low Three-dimensionality, and Rigidity, further codified as “eNTRy rules”^41^. Accumulation data highlighted the contribution of charge in accumulating compounds in *E. coli*. Compounds with a positive charge were more likely accumulated as compared to other compounds. Further investigation identified the important contribution of primary amines to compound accumulation, as compared to secondary, tertiary, or quaternary amines, since most of the accumulating compounds were found to contain primary amines. Therefore, structure-activity relationship (SAR) studies were conducted to validate the role of primary amines by displacing them with other functional groups in different classes of accumulating compounds. However, the primary amine was not the only factor contributing to compound accumulation as from the total of 68 compounds containing primary amines tested, 32 primary amines did not show accumulation. Therefore, Hergenrother and group investigated other factors essential for drug accumulation using a set of 68 primary amines which were studied for a total of 297 molecular descriptors by computational analysis. Additionally, high rigidity (as described by the number of rotatable bonds) and low three-dimensionality (as measured by a computed globularity score) of compounds seem to contribute to accumulation in *E. coli*. The probable reason for increased drug accumulation was the lower susceptibility of rigid and planar compounds to the action of efflux pumps^41^. Based on the observed results, guidelines for compound accumulation in *E. coli* were postulated. The major advantage of these chemical traits was that these parameters can be implemented by medicinal chemists into new compounds. Additionally, the compound’s amphiphilic moment and amine steric hindrance were also found to influence drug accumulation. In some cases, guanidiniums and *N*-alkylpyridiniums were also found to facilitate compound accumulation in *E. coli*^41^. Gram-positive specific antibacterial compounds optimized to broaden their anti-microbial spectrum by applying eNTRY rules are described in Table 3.

**Table 3:** List of Gram-positive specific antibacterial compounds optimized to broaden their anti-microbial spectrum by applying eNTRY rules^41,171^.

Compounds & Ref	Structures	Characteristics	
		Original compound	Modified compound with primary amine
Penicillin G Ampicillin^172^		RB = 4 Glob = 0.04 Activity limited to Gram-positive bacteria	RB = 4 Glob = 0.12 Broad-spectrum activity
REDX04139 REDX05931^173^		RB = 2 Glob = 0.07 Activity limited to Gram-positive bacteria	RB = 3 Glob = 0.05 Broad-spectrum activity
Deoxynybomycin (DNM) 6DNM-NH3^41,174^		RB = 0 Glob = 0.04 Activity limited to Gram-positive bacteria^175,176^	RB = 1 Glob = 0.09 Broad-spectrum activity
Debio-1452 Debio-1452-NH3^41,177^		RB = 4 Glob = 0.09 Activity limited to Gram-positive bacteria	RB = 4 Glob = 0.06 Broad-spectrum activity
Ribocil C Ribocil-C-PA^41,178^		RB = 5 Glob = 0.1 Activity limited to Gram-positive bacteria	RB = 6 Glob = 0.05 Broad spectrum activity
Pyrimidoindole 812 Pyrimidoindole 799^171,179^		RB = 4 Glob = 0.04 Activity limited to Gram-positive bacteria	RB = 4 Glob = 0.05 Broad spectrum activity
Hydroxamic acid 1 Hydroxamic acid 2^171^		RB = 8 Glob = 0.06 Activity against Gram-positive bacteria and efflux pump deficient *P. aeruginosa*	RB = 5 Glob = 0.04 Activity even against wild-type Gram-negative bacteria
AN3016 AN3365^171^		RB = 4 Glob = 0.08 No activity against Gram-negative bacteria	RB = 5 Glob = 0.08 Activity against clinical isolates of Gram-negative bacteria
Tetrahydropyran 4 Tetrahydropyran 21^171^		RB = 7 Glob = 0.16 Limited activity towards Gram-negative bacteria	RB = 7 Glob = 0.15 Enhanced activity against Gram-negative bacteria

A novel fluoroquinolone, delafloxacin, was designed for the treatment of acute bacterial skin and skin structure infections caused by both Gram-positive and Gram-negative bacterial pathogens. Three key structural features of delafloxacin include the lack of a strong basic group at position C7 (making the compound anionic at physiological pH), the presence of a chlorine atom in position C8 (stabilizing the heterocycle) and an aromatic ring at N1 (increases the molecular surface). The presence of the carboxylic acid in delafloxacin leads to a higher accumulation of compound at acidic pH, which is physiologically relevant in acute bacterial skin and skin structure infections. Delafloxacin has further shown good potency in treating respiratory, urinary, and sexually transmitted infections^42^.

### OM disruption as a strategy to increase permeabilization

Pentamidine is a clinical antiprotozoal drug that is used in the treatment of *Pneumocystis pneumonia*^43,44^, trypanosomiasis, and leishmaniasis^45^. It is known to have a moderate anti-bacterial activity against Gram-positive bacteria^46,47^. In addition, it potentiates novobiocin, erythromycin and rifampicin against Gram-negative bacteria, including wild-type and polymyxin-resistant strains of *A. baumannii*^48,49^. Based on the structure of pentamidine, Wesseling and group synthesized similar bis-amidine analogs^50^. SAR studies highlighted the effect of chain length, rigidity, and hydrophobicity of the linker units in bis-amidines in potentiating Gram-positive antibiotics. Long, hydrophobic linkers were generally found to be associated with non-specific membrane disruption, along with strong hemolytic activity. In contrast, linker motifs with a single aromatic ring displayed enhanced synergistic activity compared to pentamidine, without being hemolytic. Furthermore, the relative positioning of the benzamidine groups on the aromatic linker and ortho-, meta- and para-substitution pattern of the amidine moieties themselves, played an important role in potentiating the activity of erythromycin, rifampicin and novobiocin against a number of *E. coli* strains including polymyxin-resistant and carbapenem-resistant variants with MICs being reduced by factor 33- to ≥60. Additionally, bis-amidines displayed a strong synergistic activity with antibiotics targeting Gram-positive bacteria against other Gram-negative bacterial pathogens including *A. baumannii, K. pneumoniae* and *P*. *aeruginosa*. Mechanistic studies revealed that these compounds act by disrupting the OM of Gram-negatives, thus increasing the antibiotic’s permeability^9,50^.

Zhou et al. screened a library of 170 FDA-approved antibiotics in combination with pentamidine. Pentamidine was found to increase the susceptibility of *E. coli* K12 for most fluoroquinolones, tetracyclines, amphenicols, pleuromutilins, ansamycins, macrolides, oxazolidinones and a small fraction of β-lactams and nitrofurans. However, aminoglycosides, aminocoumarin, sulfonamides and lincosamide classes of drugs were poorly boosted. Further, pentamidine was found to potentiate minocycline, linezolid, valnemulin and nadifloxacin against all the standard laboratory strains of *A. baumannii*, *P. aeruginosa*, and *K. pneumoniae*. Moreover, tetracyclines and oxazolidinones displayed a strong synergistic effect while aminoglycosides displayed a strong antagonistic effect with pentamidine. Molecular descriptor-based approaches to understand the physicochemical properties of synergistic antibiotics revealed six descriptors describing four molecular characteristics, namely hydrophobicity, partial charge, rigidity and surface rugosity^51^. These descriptors are in continuation with the eNTRy rules described above. For example, the potent Gram-positive antibiotic linezolid possesses high hydrophobicity and rigidity. This was further justified by a stable MIC of the drug even with the addition of exogenous phosphatidylethanolamine (PE)^51^.

The safety of primary amine-containing compounds was questioned due to the increased risk of hERG (human ether-a-go-go-related gene) inhibition and a very limited number of commercially available antibiotics possess a primary amine group in their structure. However, the lack of primary amines in most of the commercially available antibiotics was justified by difficulties in the synthesis and purification of primary amine-containing compounds. Richter and colleagues studied and reported the safety pharmacology profiles of several primary amine-containing compounds including commercially available antibiotics β-lactams (ampicillin, amoxicillin, ceftolozane, cefadroxil, cephalexin etc.), aminoglycosides (kanamycin, amikacin, sisomicin, netilmicin etc.), vancomycin, daptomycin, colistin and many antibiotics at their discovery phase (nemonoxacin, plazomicin, surotomycin, cefilavancin, cefamulin, LTX-109, ACHN-975, epetraborole, and murepavadin)^9,43^.

### Limitations of eNTRy rules and other identified physicochemical identifiers

**Fig. 1:**
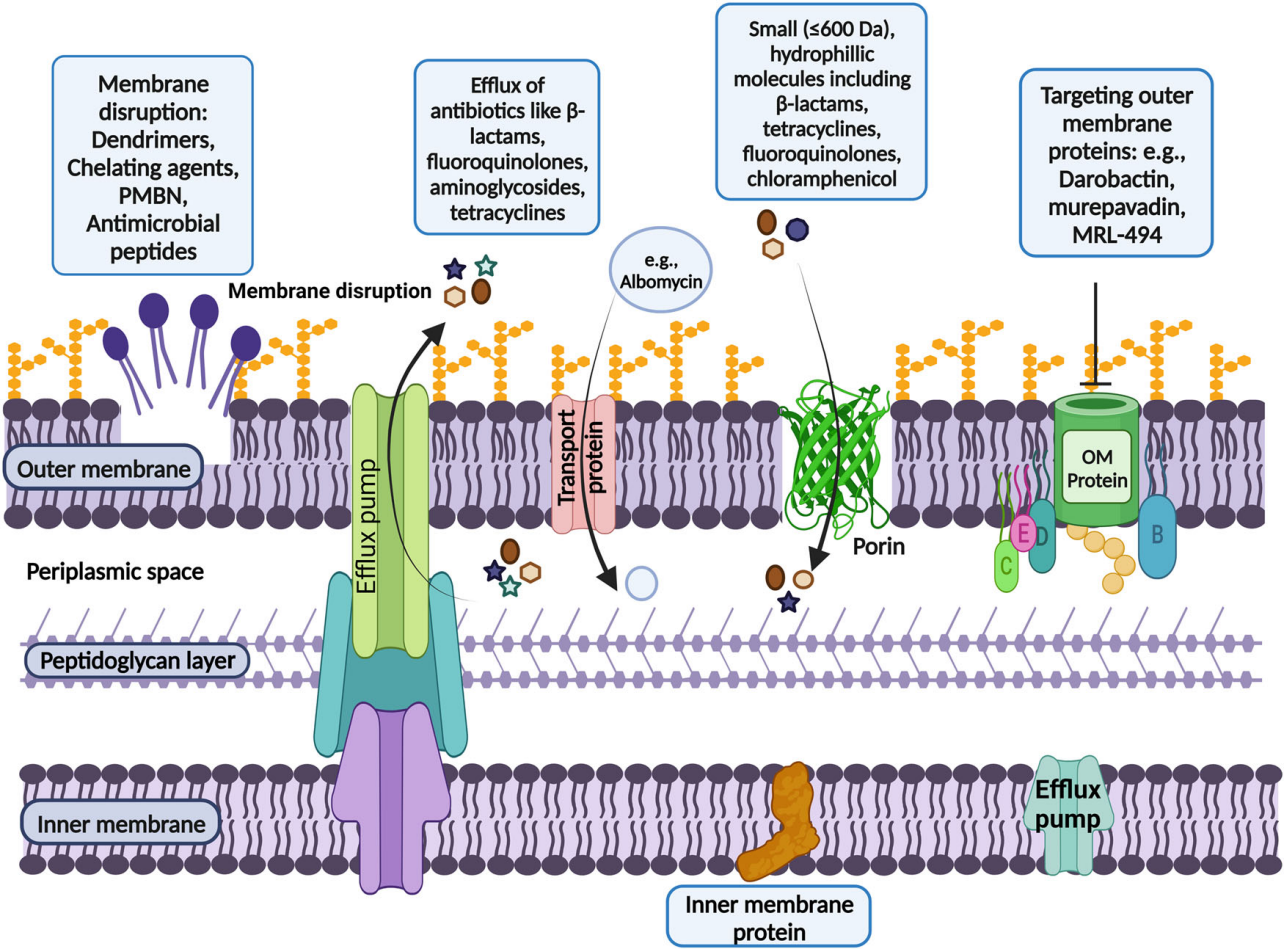
Entry routes of a wide range of antimicrobials via outer membrane of Gram-negative bacteria. The figure represents the complex architecture of the cell envelope of Gram-negative bacteria which acts as a barrier for antimicrobial agents to enter the cell because of their specific physio-chemical composition. The figure illustrates the routes of entry followed by a wide range of antimicrobials to enter the outer membrane. The ways include membrane disruption by competing with divalent cations of membrane (as followed by agents such as PMBN, dendrimers, anti-microbial peptides), hydrophilic β-barrel proteins called porins (as followed by fluoroquinolones, β-lactams, tetracyclines, chloramphenicol), targeting outer membrane proteins (e.g.,darobactin, murepavadin, MRL-494). Further, the antibacterials such as macrolides utilizes membrane diffusion mechanism to enter the bacterial outer membrane. Adding on to the entry routes, antibacterials such as albomycin make use of transport proteins to cross outer membrane of bacterial cell. However, the accumulation of antimicrobials inside bacterial cells is compensated by the presence of outer membrane efflux pumps that export the entered antimicrobial agents and other foreign substances out of the cell. Figure created with BioRender.com.

**Fig. 2:**
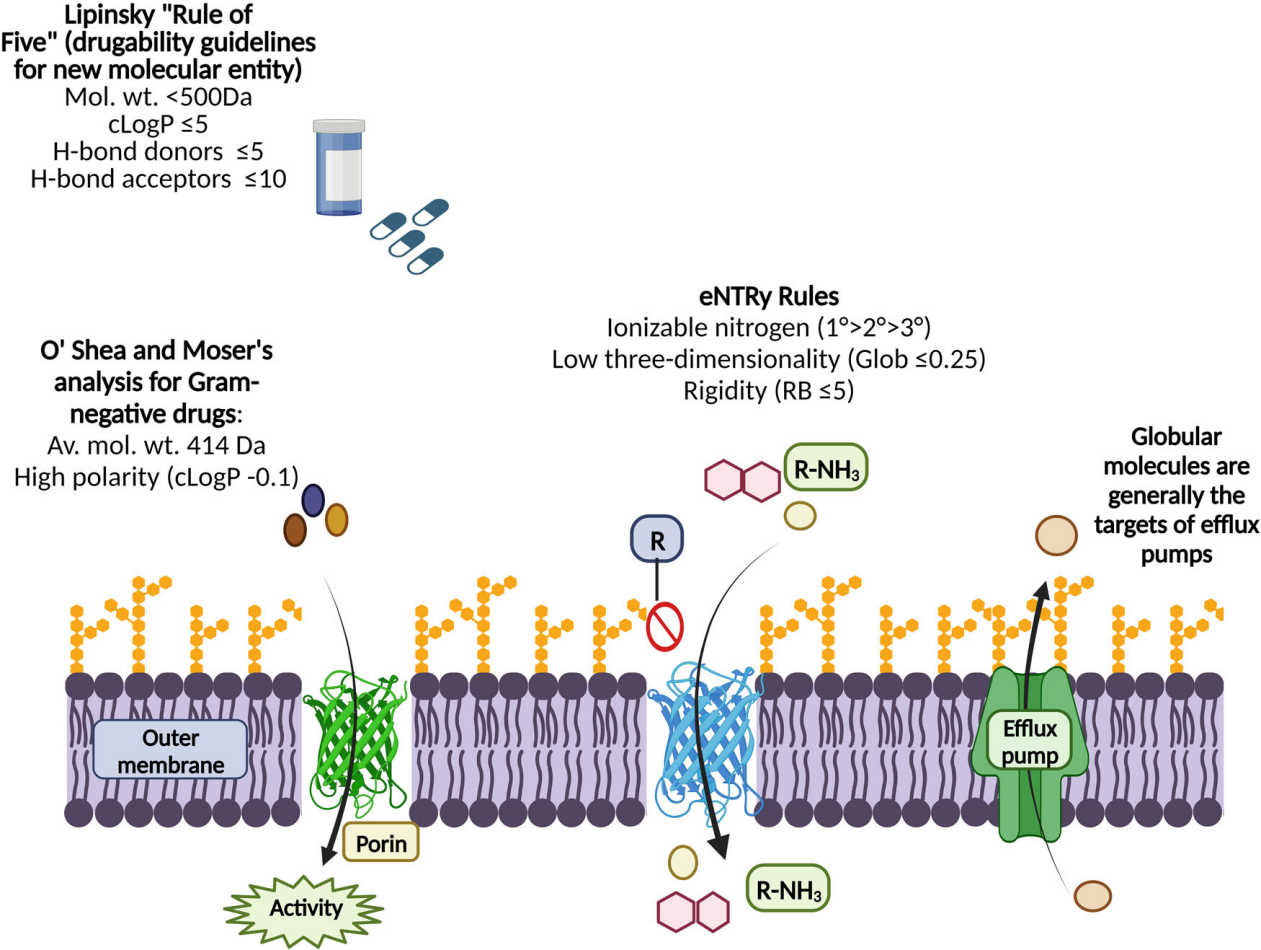
The figure illustrates the physicochemical parameters of compounds studied to overcome the outer membrane barrier of Gram-negative bacteria. Of these rules, traditional drug space of orally bioavailable compounds (Lipinski rules) suggests that a druggable molecule should possess an av. mol. wt. of <500 Da, with <5 hydrogen bond donors, ≤10 hydrogen bond acceptors and calculated octanol-water partition coefficient (clogP ≤ 5). These physicochemical properties were further narrowed down by O’Shea and Moser’s, according to which, a Gram-negative targeting compound must possess an average mol. wt. of 414 Da with clogP ~ 0.1. Later, according to the eNTRy rules proposed by Hergenrother and colleagues, compounds with ionizable nitrogen, globularity ≤0.25 and rotational bond ≤5 are more likely to accumulate in Gram negatives. Glob globularity, RB number of rotatable bonds. Figure created with BioRender.com.

Although the presence of a primary amine was in several examples found to be an important factor in promoting drug accumulation in Gram-negative bacteria, other structural features such as flexibility and shape have an important contribution in the uptake of compounds via self-promoted cell uptake. Apart from these parameters, charges present on the compounds play an important role in transporting molecules via porins for small, hydrophilic molecules and OM disruption for lipophilic and positively charged molecules. Figure 1 illustrates the mode of entry of a wide range of antimicrobials through the outer membrane components in Gram-negative bacteria e.g., porins, transport proteins and OM proteins. However, the limited accumulation and efflux of antimicrobials in Gram negative bacteria tags Gram-negative bacteria-a difficult-to-treat-microbe. In this scenario, Fig. 2 describes the rules governing the physicochemical parameters of antibacterial agents to improve their accumulation in Gram-negative bacteria.

**Fig. 3:**
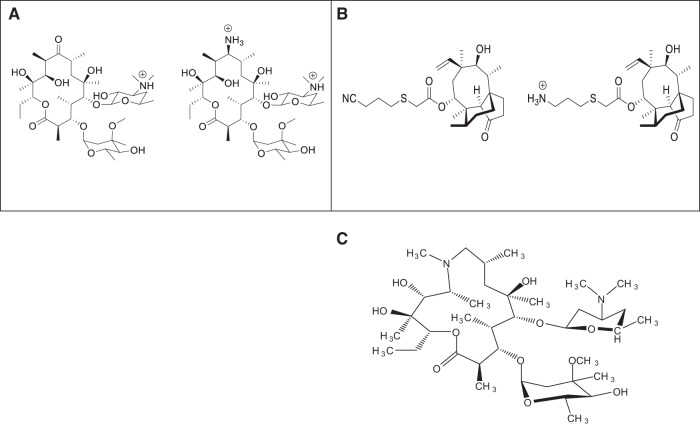
Examples illustrating limitations of eNTRy rules of broadening the antibacterial spectrum of antibiotics. **A** Erythromycin, a macrolide class antibiotic, was modified to 9(S)-erythromycyclamine to improve antibacterial spectrum by introducing primary amine to the structure. In contrast to the eNTRy rules, introduction of primary amine does not make any difference in the anti-bacterial spectrum of the compound. **B** Similarly, modification of Pleuromutilin to Pleuromutilin-amine by introduction of primary amine was not effective in improving its antibacterial activity. **C** Azithromycin, a macrolide class antibiotic, having RB=7 is studied to enter Gram-negative bacteria by self-promoted uptake, which is again in contrast to the eNTRy rules where RB of the compound to accumulate in Gram-negatives should ≤5.

An example where one of the modifications made was the addition of a primary amine that does not broaden the antibacterial spectrum was the conversion of erythromycin to (9S)-erythromycylamine^52,53^. In contrast, azithromycin, a macrolide with RB = 7; Glob = 0.24 was found to enter Gram-negative bacteria via the self-promoted pathway^54^. The structures of erythromycin and pleuromutilin along with their modified analogs are depicted in Fig. 3.

Hirsch and colleagues evaluated the applicability of eNTRy rules for repositioning an antimalarial chemical class targeting *Plasmodium falciparum* (Pf) IspE, which lacked activity against bacterial pathogens even in the presence of PMBN. They designed and synthesized a library of amino acid-modified compounds together with the respective Boc-protected analogs. Of these analogs, only glycine-containing compounds were found to show activity against *E. coli* while suffering efflux issues^55^. Taken together, there are several physicochemical factors that affect the entry and accumulation of compounds in Gram-negative bacteria. These parameters alone or in combination need to be analyzed for developing broad-spectrum antibiotics against MDR Gram-negative bacterial pathogens.

### Other strategies targeting the outer membrane of Gram-negative bacteria

In addressing the problem of OM permeability in Gram-negative bacteria, a number of new and innovative approaches are currently being investigated. The strategies include interfering with LPS biosynthesis, targeting OM proteins such as the β-barrel assembly machine (BAM) complex, entry of compounds via siderophores via a Trojan horse strategy, co-administration of drugs with membrane disrupting molecules or efflux pump blockers^9^. Details of these strategies are discussed in the following section.

#### Targeting outer membrane proteins

OM proteins are attractive and easy-to-approach targets for antibacterial drugs^56^. Drugs targeting these proteins indirectly lead to OM disruption. The acetyltransferases LpxA and LpxD are involved in the first and third steps respectively of LPS biosynthesis in Gram-negative bacteria. Both these enzymes are essential for bacterial growth^56^. New England Biolabs screened a library of ~1.9 billion random 12-amino acid peptides, fused to M13 phages. Peptide RJPXD33 (TNLYMLPKWDIP) was found to exhibit moderate affinity with LpxD (*K*_d _= 6 µM). Additionally, the compound was also found to bind and inhibit LpxA (*K*_d_ = 22 µM). RJPXD33 contains a conserved fatty acid binding groove similar to LpxA and LpxD, thus highlighting its dual-target potency. Although no whole-cell activity was reported for RJPXD33, the property of the compound to target essential proteins opens new drug targets for drug discovery^56,57^.

Similarly, other enzymes involved in lipid biosynthesis, Kdo biosynthesis, and LPS transport were studied to identify new potential targets for drug discovery^56^. A list of compounds under study aiming LPS disruption or targeting the outer membrane proteins of Gram-negative bacteria is mentioned in Table 4.

**Table 4:** List of compounds, under study, aiming LPS disruption or targeting outer membrane proteins of Gram-negative bacteria.

S. No	Compound	Structure	Developmental stage	Target (Target location)	References
Compounds targeting LPS biosynthesis					
1.	RJPXD33		Pre-clinical trials	LpxA/LpxD (Cytoplasm)	^57^
2.	L-573,655		Pre-clinical trials	LpxC (Cytoplasm)	^180^
3.	ACHN-975		Completed Phase I Clinical Trial	LpxC (Cytoplasm)	^181,182^
4.	PD 404182		Pre-clinical trials	KdsA (Cytoplasm)	^183^
5.	MAK-181	undisclosed	Pre-clinical trials	LpxC (Cytoplasm)	^184^
6.	Myxovirescin (TA)		Pre-clinical trials	LspA (Inner membrane)	^185,186^
7.	Globomycin		Pre-clinical trials	LspA (Inner membrane)	^187^
Compounds targeting LPS transport					
8.	G907		Pre-clinical trials	MsbA (Inner membrane)	^188^
9.	Novobiocin derivatives		Pre-clinical trials	LptB (Inner membrane peripheral protein)	^189^
10.	Murepavadin, POL7080		Phase III clinical trial	LptD (Outer membrane)	^190,191^
11.	Thanatin		Pre-clinical trials	LptA and LptD (Periplasm and outer membrane respectively)	^192^
12.	4-phenylpyrrolocarbazole		Pre-clinical trials	LptB and LptB_2_FGC IM complex (Inner membrane)	^185,193^
13.	G0507		Pre-clinical trials	LolCDE (Inner membrane)	^194^
14.	MAC13243, A22		Pre-clinical trials	LolA (Periplasm)	^195–197^
Compounds targeting BAM complex					
15.	MRL-494		Pre-clinical trials	BamA (Outer membrane)	^198^
16.	BamA-derived peptide fragment SAGIALQW		Pre-clinical trials	BamD (Outer membrane)	^199^
17.	Darobactin		Pre-clinical trials	BamA (Outer membrane)	^60^
18.	POL7306 A β-hairpin macrocycle linked to polymyxin, (Unspecified amino acid residues are indicated by an X)		Pre-clinical trials	BamA and LPS (Outer membrane)	^200,201^

García-Quintanilla and coworkers have studied the efficacy of the LpxC inhibitor PF-5081090, which at 32 µg/mL effectively potentiates rifampin, vancomycin, azithromycin, imipenem and amikacin against MDR *A. baumannii* strainsleading to significant increase in cell permeability^58^.

Vinuesa et al. studied the synergistic effect of LpxC inhibitors with iron chelators, 2,2′-bipyridyl (BIP) and deferiprone, and gallium nitrate (Ga(NO_3_)_3_) against *A. baumannii* including MDR strains. LpxC inhibitor LpxC-2 displayed synergy with BIP against 30% of strains tested (FICI values: 0.38–1.02), and with Ga(NO_3_)_3_ against 50% of tested strains (FICI values: 0.27–1.0). Another LpxC inhibitor, LpxC-4 displayed potent synergy with Ga(NO_3_)_3_ against all the tested strains with FICI values: 0.08– ≤ 0.50^59^.

Imai and group studied the antibacterial property of 67 strains of nematode symbionts*Photorhabdus* and *Xenorhabdus*against *E. coli*. A small zone of *E. coli* inhibition was seen for *P. khanii* HGB1456. Bioassay-guided isolation of bacterial extracts led to the identification of a modified heptapeptide, darobactin (W^1^-N^2^-W^3^-S^4^-K^5^-S^6^-F^7^ with two cycles formed between W^1^-W^3^ and W^3^-K^5^). Darobactin significantly inhibits MDR *E. coli*, *K. pneumoniae*, and some strains of *P. aeruginosa*, including polymyxin-resistant, ESBL (extended-spectrum β-lactamase), and carbapenem-resistant clinical isolates at 2–4 μg/ml. The compound was found to be bactericidal against *E. coli* with a minimum bactericidal concentration (MBC) of 8 μg/ml. Additionally, the compound was not cytotoxic against HepG2, FaDu and HEK293 cell lines with IC_50_ > 128 μg/mL. Sequencing of darobactin-resistant mutants highlighted 2–3 mutations in the OM essential protein BamA, the core protein of the BAM machinery. By targeting BamA, darobactin disrupts the complete β-barrel assembly machinery (BAM) of GNB, thereby disrupting the folding and assembly of OM proteins^60^. Müller and group designed and biosynthesized new darobactin analogs based on cryo-EM structure of BamABCDE (BAM)-D9 complex. Analogs were generated by the core peptide modification strategy, which includes addition of multiple halogenated tryptophans. Of these derived analogs, darobactin 22 (D22) was found effective against clinical strains of carbapenem-resistant *A. baumannii* and *P. aeruginosa* with up to 32- and 4-fold reduction in MIC values. In fact, the compound was equipotent to colistin^61^.

#### Efflux pumps inhibitors (EPIs)

EPs play a predominant role in the development of intrinsic resistance among microbes rendering them attractive targets in the field of antibacterial therapy. Most antibiotics that penetrate the OM or reach the cytosol of Gram-negatives cells are ejected by these efflux pumps. They prevent the accumulation of compounds within the periplasmic space and the bacterial cytosol, thus protecting the bacteria from the action of an anti-bacterial agent^12^. Several studies have shown that the antibacterial activity of a drug molecule is restored in efflux pump deficient/mutated bacteria.

In this scenario, the development of efflux pump inhibitors was initiated to rehabilitate the activity of antibiotics in resistant strains. Among the different ways of inhibiting the activity of efflux pumps, two possible ways are by blocking the synthesis of ATP via stopping the formation of the electrochemical proton gradient, or by preparing antibiotic-competing compounds to prevent their excretion. Examples of competitive RND efflux pump inhibitors comprise the dipeptidyl amide phenylalanine-arginine-β-naphthylamide (PAβN), pyridopyrimidines, and arylpiperazines. Among the arylpiperazine inhibitors, 1-(1-naphthylmethyl)-piperazine (NMP) is the most effective^62^. Yoshida and colleagues developed the derivative D13–9001, which specifically inhibits the MexAB-OprM pump and displays good in vitro activity against *P. aeruginosa*^63^. Similarly, hydantoin derivatives were found to restore antibacterial potency of antibiotics like nalidixic acid and chloramphenicol against *Enterobacter aerogenes* by inhibiting the AcrAB–TolC system^64^.

To date, a number of efflux pump inhibitors have been studied and synthesized to potentiate antibiotics against MDR Gram-negative bacterial pathogens^65^. Although efflux pump inhibitors are an attractive strategy to increase drug accumulation, their development faces several roadblocks. Commercial production of naturally derived efflux pump inhibitors finds difficulty in synthesizing molecules because of their complex structures, while synthetic inhibitors face poor solubility and toxicity issues. Moreover, being used as a combinational therapy, pharmacokinetic compatibility of both the drugs is required. Additionally, resistance mechanisms other than efflux limit the implication of antibiotic-efflux pump inhibitor combinational therapy. Generally, the efflux pump inhibitor binds at a specific site on target, thus limiting the number of substrates it acts upon. This was observed with PAβN which potentiates only a limited set of antibiotics while being ineffective for others, thus, limiting the use of this combinational therapy. Therefore, antibiotic-efflux pump inhibitor combinations require more studies to develop effective therapy strategies against drug-resistant bacterial pathogens^66^.

#### Synergy: a cooperative way of transporting a compound over the OM

The development of membrane disruptive agents offers a synergistic effect with Gram-positive selective antibiotics and potentiates them towards Gram-negative bacteria. In the field of synergy, a fractional inhibitory concentration index (FICI) below 0.5 is considered as a benchmark for the synergistic activity^67^.

Polymyxin B was the first and very effective membrane-disrupting lipopeptide against Gram-negative bacteria. However, nephrotoxicity associated with polymyxin B restricts its use^68^. Polymyxin-resistant strains show changes in the LPS layer of the OM, a reduced membrane negative charge and a reduced number of porins^69^. Therefore, Polymyxin B nonapeptide (PMBN), the first and most effective membrane-disruptive synergist against Gram-negative bacteria, was designed by removing the lipid moiety of polymyxin B and is used as a benchmark for synergistic activity. The compound itself does not possess any antibacterial activity, but effectively potentiates the entry of other antibiotics^22^. After the discovery of PMBN as a potent permeabilizing synergist of Gram-negative bacterial OM, a vast amount of research has been done on synergists that being inactive alone, can potentiate other antibiotics against Gram-negative bacteria^9^.

#### Peptide-based synergists

Apart from PMBN, several truncated derivatives of polymyxin B, including deacylpolymyxin B (DAPB), polymyxin B octapeptide (PMBO) and polymyxin B heptapeptide (PMBH), were found to elicit synergy against Gram-negative bacteria with limited toxicity^22^. SPR741 (formerly, NAB741), a PMBN derivative with only three positive charges and no lipophilic tail was found to display a potent synergistic effect^70^. The compound has successfully completed a phase I clinical trial and is now being investigated further for its in vivo efficacy. Additionally, it has been reported that increasing the lipid chain diminishes its anti-bacterial potency but enhances its synergistic potential^9^. Examples of such compounds are NAB739 and NAB7061, where NAB739 is a very dominant synergist but has less inherent antimicrobial activity than NAB7061^57,71,72^. Several endogenous peptides in humans and other mammals associated with natural innate immunity are found to target the OM of Gram-negative bacteria. Therefore, a large number of derivatives targeting OM of Gram-negatives were designed based on natural human peptides such as cathelicidins, lactoferrin, thrombin, novicidin (sheep), bactenectin (bovine) and indolicidine (bovine). These peptides can be used in combination with drugs that can naturally not be transported in GNB^9^.

A similar membrane targeting effect was seen for *S*-(3,4-dichlorobenzyl) isothiourea (A22) and its analogs, that target the bacterial cytoskeletal protein MreB, thereby resulting in increased membrane permeability^73^.

In the field of peptide-based synergy, lipopeptide-based compounds have been studied extensively. Although they do not possess any antibacterial activity, they can enhance the activity of other antibiotics. They carry a few unnatural and D-amino acids and are designed based on peptides produced by *Paenibacillus sp*. strain OSY-N. These lipopeptides show low hemolytic activity, exhibit potent synergy by possessing OM-disrupting activity and are found to potentiate clarithromycin and rifampin against polymyxin-resistant *E. coli* in a murine thigh infection model. Another example of a lipopeptide, paenipeptin-inspired synergist (SLAP-S25), was found to boost the activity of rifampicin and vancomycin against *E. coli*. SLAP-S25 is established to have disruptive effects on both outer and inner membranes, which is proven by its binding to LPS and phosphatidylglycerol (PG)^74^.

Dilipid ultrashort cationic lipopeptides (dUSCLs) are lysine-rich tetrapeptides with various lipids at the N-terminal of the peptides. Such dUSCLs are found to have the capability of enhancing the activity of clinically used antibiotics against Gram-negative bacteria^75^.

Besides, a series of ultrashort tetrabasic lipopeptides with three basic amino acids separated by a molecular scaffold bis(3-aminopropyl)glycin, along with simple fatty acids is also reported to be capable of potentiating clinically used antibiotics against Gram-negative bacteria, which are previously selective for Gram-positive bacteria only^76^.

#### Antibiotic-adjuvant combination approach

An adjuvant is a bioactive helper molecule that enhances the activity of antibiotics. It can be an efflux pump inhibitor, a membrane permeabilizer or an enzyme inhibitor. An antibiotic-adjuvant combination approach is one of the most successful forms of treatment therapy where an antibiotic is given in combination with a helper molecule. The activity of this kind of synergist such as a β-lactam–β-lactamase inhibitor combination (e.g., amoxicillin and clavulanic acid, ceftolozane-tazobactam, ceftazidime-avibactam and meropenem-vaborbactam, Imipenem-cilastatin-relebactam triple combination, Aspergillomarasmine A-meropenem) was explored and was found to overcome antibiotic-resistance mechanisms, as reviewed previously^77^.

Some antibiotic-derived synergists are also explored where antibacterial compounds having OM disruptive activity are conjugated with antibiotics that are otherwise inactive towards Gram-negatives. Aminoglycosides like tobramycin have OM disruptive capability via binding to LPS, thereby causing membrane depolarization. A series of tobramycin-fluoroquinolone conjugates such as tobramycin-moxifloxacin^78^ and tobramycin-ciprofloxacin^79^ conjugates were explored and found to enhance the antibiotic activity of several reported drugs, including rifampicin, erythromycin, novobiocin and vancomycin against Gram-negative bacteria. Tobramycin was also found to improve the efficacy of lysine-based amphiphiles by permeabilizing the bacterial outer membrane^80,81^. These lysine-based amphiphiles include, amongst others, erythromycin and vancomycin. A combination of novobiocin and a tobramycin homodimer has shown efficacy against *A. baumannii* in a wax moth larvae model^82^. Tobramycin was also explored after coupling with small-molecule efflux pump inhibitors such as 1-(1-naphthylmethyl)piperazine (NMP) and paroxetine. These inhibitors were found to potentiate its activity against *P. aeruginosa*^83^. These synergists are also found to cause OM disruption and inner membrane depolarization. A novel hybrid of levofloxacin and a polybasic peptide was found to exhibit strong activity against MDR clinical *P. aeruginosa*, *E. coli*, *K. pneumoniae* and *A. baumannii*^84^.

Recently, two generations of *P. aeruginosa* biofilm-targeted conjugates of fluoroquinolone antibiotics and glycomimetic lectin binders were disclosed. Introduction of a peptide-based prodrug mechanism reduced the toxic potential of the attached drugs while retaining full antibiotic activity after self-destructive release by *P. aeruginosa*^85,86^.

#### Antibiotic hybrid approach

The antibiotic hybrid approach fuses two or more antibacterial agents into one heteromeric entity, ideally retaining the activity of the individual constituent fragments. This linkage of fragments can be cleavable (prodrug approach) or non-cleavable (hybrid drug approach) and occur between two antibiotics or antibiotic and adjuvant^77^. For example, an antibiotic hybrid was generated by linking a cefamandole (cephalosporin) derivative to omadine (a metal chelator and bacterial ATP synthesis inhibitor) to form a hybrid prodrug^87^. Most of the antibiotic hybrid prodrugs are cephalosporin-based. Some other examples of antibiotic hybrids are desacetylcephalothin linked to the alanine racemase inhibitor chloroalanyl dipeptide^88,89^, desacetylcephalothin linked to triclosan-NB2001^90^, and desacetylcefotaxime linked to fleroxacin-Ro 23–9424^91^. These hybrid prodrugs displayed potent activity against a panel of Gram-negative bacterial pathogens.

**Fig. 4:**
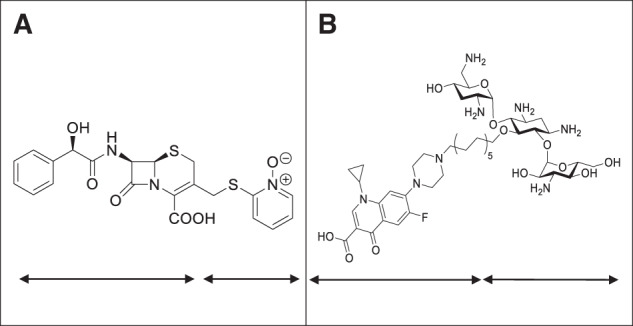
Structures of commonly studied antibiotic hybrids. **A** Cefamandole-omadine conjugate, a cephalosporin and 2-mercaptopyridine-*N*-oxide or pyrithione (metal chelator and ATP synthesis inhibitor) conjugate and **B** ciprofloxacin‑tobramycin conjugate that effectively targets Gram-negative bacteria via dual mode of action.

Tobramycin-based hybrids with fluoroquinolones were developed to promote the entry of antibiotics into the periplasm of Gram-negative bacterial cells by self-promoting the uptake mechanism of aminoglycosides (see above). These hybrids displayed antimicrobial activity against *P. aeruginosa*^77^. Antibiotic hybrids are thus an interesting approach to drug discovery, but require certain points into consideration: 1. Linking of two moieties should be at non-pharmacophoric regions to ensure the retention of biological activity of both compounds. 2. Spatial length of linker should be optimized^77^. Structures of cefamandole-omadine conjugate and tobramycin-ciprofloxacin conjugate are depicted in Fig. 4.

#### Chelating agents as OM-disrupting synergists

OM-disrupting molecules such as chelators or polymyxins compete with divalent cations cross-bridging neighboring LPS molecules. Displacement of these ions leads to enhanced lateral diffusion of molecules in the bacterial membrane^6^. The potential of the chelating agent EDTA to synergize with other antibiotics against Gram-negative bacteria is well known^92^. Additionally, Ayres and Russell have highlighted the ability of sodium polyphosphates as potent synergists with several antibiotics^93^. Citric acid is also found to exhibit synergistic activity with antibiotics including erythromycin, novobiocin, rifampicin, methicillin, and gentamicin^93^. Moreover, 2,3-dimercaptosuccinic acid, an effective treatment of lead intoxication, was also found to potentiate hydrophobic antibiotics against Gram-negative bacteria by permeabilizing OM of bacteria, as depicted by increased N-phenylnaphthylamine (NPN) uptake^9^. Despite the excellent activity of chelating agents, polymyxin-resistant strains of *S. typhimurium* and *E. coli* were also reported with 75% lower binding to polymyxins compared to the parent strains. Studies conducted on the LPS layer of these bacteria revealed increased levels of aminoarabinose and phosphoethanolamine formation due to the esterification of lipid A phosphates. This lowered negative charge significantly reduces the binding of polymyxin and other chelating agents^6^.

### Terpene-based synergists

Some natural products possess antibacterial activity due to their membrane permeabilizing ability and show synergistic effects with antibiotics against MDR Gram-negative bacteria. For example, some terpenoids such as eugenol, citronellol, carvacrol, geraniol, menthol, myrcene, and thymol show synergistic effects with penicillin against *E. coli*. Eugenol, a compound found in clove oil from *Eugenia aromatic* functions as membrane permeabilizer via inhibiting an ATPase and has synergistic effect with ampicillin, penicillin, oxacillin, erythromycin, norfloxacin, tetracycline, chloramphenicol, vancomycin, rifampin, and polymyxin B against MDR Gram-negative bacteria, following different mechanisms. Among these synergized antibiotics, penicillin, ampicillin, oxacillin, vancomycin, and polymyxin B affect the cell membrane via different targets, norfloxacin damages the cell membrane by removing divalent cations from LPS-binding sites, while erythromycin, rifampin, tetracycline, and chloramphenicol attack the ribosome of bacteria^94^.

#### Entry via siderophores and their mimetics

Iron, under physiological conditions, is scarcely present in its free form (10^–9^ M) due to its rapid oxidation from Fe^2+^ to Fe^3+^, subsequently forming insoluble hydroxides. The uptake of iron in this form is a challenge for organisms relying on this naturally available iron. To circumvent iron shortage, bacteria display certain receptor proteins that bind a variety of iron-containing molecules. Additionally, they produce and secrete high-affinity iron-chelating compounds called siderophores. Upon binding to bacterial receptors, they are translocated across the membrane via ABC importers^95^. A natural Trojan horse mechanism of delivering antibiotics along with siderophores was identified in bacteria through the identification of albomycins (ferrichrome siderophore analog conjugated with thioribosyl pyrimidine antibiotic), ferrimycins, salmycins and some microcins. These siderophores were found to show antibacterial effects against several Gram-negatives. For example, albomycin (secreted by *Streptomyces* strains) displays activity against certain GNB including *E. coli*. Similarly, microcin E492 (MccE492) produced by *K. pneumoniae* RYC492 displayed activity against *E. coli*, *S. enteritidis* and *S. typhimurium*^96^. Naturally occurring hydroxamate-type siderophores are covalently attached to the antibiotic moiety to formulate these sideromycins. Considering this transport mechanism, efforts were made to increase antibiotic-loaded sideromycin uptake in pathogenic bacteria. Generally, conjugation involves catechol/hydroxamate siderophore analogs covalently attached to a β-lactam drug^97^.

Zahner and group reported the first synthetic siderophore antibiotic conjugate in which desferrioxamine B (DFO B) with a sulfonamide was ligated to varying cephalosporines, fluoroquinolones, and other antibiotics. However, none of them was found to target Gram-negative bacteria^98^. Later, Miller and coworkers designed tris-catechol siderophore derivatives, containing an enterobactin analog linked to amipicillin/amoxycillin, that displayed potent antimicrobial activity against *P. aeruginosa*^99^.

The siderophore monosulfactam BAL30072 has demonstrated antibacterial potency against MDR Gram-negative bacteria in vitro and in vivo. BAL30072 is a combination of a siderophore-mimicking small molecule, hydroxypyridone and a monocyclic β-lactam antibiotic linked via an oxime, which enables access to the bacterial cell through the iron uptake system. Under in vitro conditions, it has displayed its inhibitory effect against MDR strains of *Burkholderiapseudomallei*, *P. aeruginosa* and *A. baumannii*, including strains producing class C carbapenemases. In an in vivo rat soft-tissue infection model, the conjugate potently inhibited 80% of *A. baumannii* strains tested. Additionally, the combination of BAL30072 and carbapenems was found effective against MDR Gram-negative bacteria, particularly against *Enterobacteriaceae* and *P. aeruginosa* in vitro. BAL30072 has been tested in a phase I study in human subjects but the current development status is unknown^97^.

**Fig. 5:**
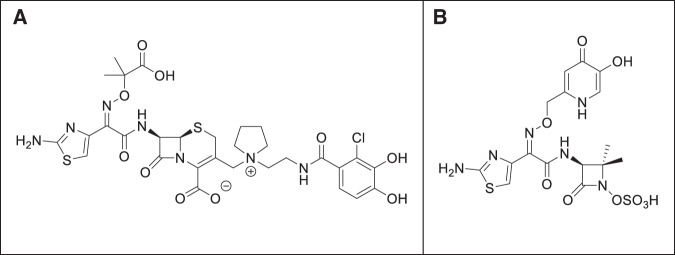
Structure of common siderophore‑antibiotic conjugates. **A** Cephalosporin conjugate cefiderocol (Fetroja), an FDA approved siderophore cephalosporin conjugate for the treatment of hospital acquired bacterial pneumonia (HABP) and ventilator-associated bacterial pneumonia (VABP). **B** Monosulfactam BAL30072, a monocyclic beta-lactam antibiotic with potent activity against drug-resistant Gram-negative pathogens.

Shionogi & Co has developed a novel siderophore cephalosporin conjugate, cefiderocol (brand name Fetroja), for treating UTI and adult pneumonia caused by MDR Gram-negative bacteria. Structurally, cefiderocol is similar to ceftazidime (third-generation cephalosporin, having a pyrrolidinium group at position 3) and cefepime (fourth-generation cephalosporins, having an oxime group at position 7) along with a catechol group on the side chain at position 3 which differs from both ceftazidime and cefepime. Its high stability made the compound active against a variety of Ambler class A, B, C, and D β-lactamases. Under in vitro conditions, it was found more potent than both ceftazidime-avibactam and meropenem against MDR strains of *A. baumannii, K. pneumoniae*, carbapenemase (KPC)-producing Enterobacterales, MDR *P. aeruginosa* and *Stenotrophomonas maltophilia*. Under in vivo conditions, cefiderocol potently inhibited MDR and XDR Gram-negative bacterial pathogens in mouse and rat infection models^100^. The compound acts by binding to penicillin-binding protein-3, thereby, inhibiting bacterial cell wall synthesis. The compound has proven its efficacy in clinical trials and was approved by FDA in 2020 for the treatment of HABP and VABP^28^. The structure of cephalosporin conjugate cefiderocol (Fetroja) and monosulfactam BAL30072 is shown in Fig. 5.

Adding to the list, artificial siderophores based on the 1,3,5-*N,N*′*,N*′′-tris-(2,3-dihydroxybenzoyl)-triaminomethylbenzene (MECAM) core were studied in conjugation with β-lactam drugs and daptomycin against Gram-positive bacteria and Gram-negative bacteria. MECAM–aminopenicillin conjugates were found to display some activity against Gram-positive and Gram-negative pathogens^101^. A variety of synthetic siderophores such as linear Orn-derived tris-hydroxamate, isocyanuric acid-based tri-Orn hydroxamate, biscatecholate-monohydroxamate, etc. have been designed to date. These siderophores share structural similarities with naturally occurring catechol and hydroxamate siderophores. Ligation of these synthetic or semisynthetic siderophores with antibiotic warheads can be an effective bullet targeting GNB^98^. However, designing siderophore-antibiotic conjugates is limited by their extracellular stability, uptake pathways, receptor binding properties, and its transport across the membrane^96^.

Although the transport of antibiotics via siderophore has proven to be an attractive antibacterial combination, its efficacy is compromised by downregulation of iron transport receptors in response to the competition between siderophore-antibiotic conjugates and native siderophore production. For example, deletion of TonB-dependent receptors PiuA and PirA in *A. baumannii*, frameshift mutations in inner membrane protein complex in *exbD3* or *tonB3 genes*, overexpression of proteins involved in regulation of iron transport proteins (e.g., FecIRA operon) were found to affect the efficacy of siderophore conjugates including BAL30072 and MC-1^28^. Additionally, several mechanisms of bacteria confering resistance to cefiderocol have now been studied and beautifully reviewed by Karakonstantis and colleagues. These mechanisms include expression of β-lactamase enzymes (including NDM, KPC, and AmpC, OXA-427, and PER- and SHV-type ESBLs), porin mutations (OmpK35, OmpK36 and OmpK37 in *K. pneumonia*, OprD in *P. aeruginosa* and OmpC, OmpF in *Enterobacter* spp.), modification in siderophore receptors (PiuA/PiuD and PirA in *P. aeruginosa*, CirA and/or Fiu in Enterobacterales, *pirA* and *piuA in A. baumannii* and cir A in *K. pneumoniae)*, over-expression of efflux pumps (sugE and chrA in *K. pneumoniae*, MexAB–OprM in *P. Aeruginosa*, smeDEF in *S. maltophilia* and AxyABM in *Achromobacterxylosoxidans*), and target (PBP-3) modifications^102,103^.

A study conducted from 2018 to 2019 across China highlighted the involvement of NDM-5, 4 amino-acid insertions (YRIN/YRIK) at position 333 of penicillin-binding protein 3 (PBP3) and expression of premature siderophore receptor *cirA*in conferring resistant against cefiderocol^104^.

#### Delivery systems of colicin-like bacteriocin

Some bacteria, such as *E. coli* and related species produce and release small antimicrobial peptides called colicins that show their lethal effect against other susceptible bacteria (e.g., *Citrobacter freundii* releases colicin A or *Shigella boydii* releases colicin U). These peptides bind to specific receptors present on OM for entry of specific nutrients and translocate these nutrients through OM and periplasm by utilizing the Tol or TonB systems. Their mechanism of action involves either the formation of a voltage-dependent channel into the inner membrane or utilizing their endonuclease activity on DNA (e.g., ColE2), rRNA, or tRNA (RNase colicins (E3, E4, E5, E6, and D)). Several other bacteria producing such bacteriocins include *Pseudomonas pyogenes* (pyocins), *Enterobacteriaceae* such as *E. cloacae* (cloacins), *Yersinia pestis* (pesticins), *Klebsiella* species (klebicins or klebocins), *Serratia marcescens* (marcescins, colicins L and 24), *Photorhabdusluminescens* (lumicins), and *Bacillus megaterium* (megacins)^105^.

Delivering a drug molecule through this pathway was studied by Biswas and colleagues. They assessed the synergistic activity of *Lactobacillus* bacteriocin extract alone and in combination with antibiotics cefotaxime, polymyxin B, imipenem, and tigecycline. Purified bacteriocin from *Lactobacillus*, *Pediococcus,* and *Enterococcus* potentially displayed significantly higher inhibition in combination with antibiotics, as compared to antibiotics alone against MDR clinical pathogens *E. coli* (GN9, IB9, GN13), *K. pneumoniae* KP7, and *A. baumannii* AB13775. The combination was even effective in inhibiting the growth of clinical pathogens harboringβ-lactamases^106^.

Additionally, lectin-like bacteriocins, such as LlpAs, were identified as midsize bacteriotoxins that include tandem B-lectin domains followed by a short non-conserved carboxy-terminal extension. These bacteriotoxins are unable to penetrate the outer membrane due to their large size but act by targeting the outer membrane protein BamA, thereby impairing the BAM machinery of Gram-negative bacteria^107^. Several bacteriocins (class II bacteriocins, nisin, other lantipeptides) are reported to be noncytotoxic on different eukaryotic cell lines even at higher concentrations. The small size, non-immunogenic nature, biocompatibility, and biodegradability of bacteriocins make them an ideal candidate to be studied further as a replacement strategy for antibiotics^108^.

#### Charge-based lipid rearrangements

Interaction of positively charged ions with negatively charged LPS results in the rearrangement of lipid molecules, creating a temporary entry pathway for compounds into the periplasmic space. Octenidine (OCT, *N,Nʹ*-(1,10 decanediyldi-1[4H]-pyridinyl-4-ylidene)-bis-(1-octanamine) dihydrochloride) enters Gram-negative bacteria via this mechanism. OCT is a synthetic antimicrobial molecule deployed in several countries for skin, mucous membrane, and wound anti-sepsis. OCT was found to display a broad-spectrum antibacterial activity against both MDR Gram-positive and Gram-negative bacteria. The compound displayed its high efficacy at a low concentration within a short contact time, even in the presence of interfering substances such as blood or mucin. MOA studies revealed that the compound interacts with the OM of Gram-negative bacteria, resulting in chaotic lipid rearrangement. This membrane disruption leads to the entry of the compound into the periplasmic space^109^.

#### Translocation through nanopores

An additional pathway of entry of specific antibiotics into bacterial cells is through bacterial nanopores. Bacterial cells utilize these nanopores to transport ions and other substrates into the cells. The conduction through these nanopores depends largely on parameters such as ion current, temperature, electrostatic and steric effects^110^. Several studies have been conducted to study the transport of cations and anions through the matrix porins OmpF, osmoporin OmpK36, phosphoporinPhoE by Brownian dynamics simulations and highlight the effect of change in electrostatic potential at pore constriction site on ion transport^111^.

Jiajun and group studied the effect of divalent ions (like magnesium) on fluoroquinolones entry into bacterial cells via OM nanopores. The study revealed that divalent ions bind to the negatively charged residues inside the porins. By doing so, they reverse the ion selectivity of the pore, thus allowing the entry of molecules which else were unable to enter. Moreover, divalent ions can also chelate with antibiotic molecules like fluoroquinolone and produce differences in dipole moment of both the compounds involved, thereby affecting entry via these pores^112^.

#### Liposome-mediated entry

Another promising strategy for delivering a drug molecule into a bacterial cell is lipid-based delivery (nano)systems. The strategy involves encapsulation of a drug molecule in liposomes, which offers stability and safety to the drugs by accumulating it at infection site, minimizing drug toxicity, and protecting it from peripheral degradation. Liposomes are vesicular concentric bilayer structures surrounding aqueous compartments or units. Their unique architecture which is capable of encapsulating hydrophilic drugs within the aqueous compartment and/or carrying hydrophobic drugs inside their lipid bilayer makes them different from other nanoparticles. Liposomes vary according to size, number of lamellae, lipid composition, charge of the bilayer (anionic, cationic, or neutral), and surface functionalization with polymers or ligands. Liposomes act on Gram-negative bacteria by fusing with their OM, leading to its structural disruption and potentially reversing its low permeability. A number of antibiotic-fused liposomes have been reported that have potentiated the otherwise inactive molecules against clinically important Gram-negative bacterial pathogens. For example, aminoglycoside-loaded liposomes targeting resistant clinical strains of *P. aeruginosa*were reported by Mugabe et al. These liposomes resulted in an increased antimicrobial susceptibility of the bacteria as compared to free drug^113^. Liposomal formulations of aminoglycosides are in clinical use. Nacucchio et al. studied the potency of liposome-encapsulated antibiotics to address resistance associated with enzymatic hydrolysis. Phosphatidylcholine and cholesterol-containing liposomes with piperacillin were able to overcome hydrolysis by exogenous staphylococcal β-lactamases^114^. Adding to the advantage, liposomes encapsulating rifabutin was found effective even against MDR intracellular pathogens like *Mycobacterium tuberculosis*. Moreover, Polymyxin B encapsulated in Chitosan–dipalmitoylphosphatidylcholine:distearoylphosphatidylethanolamine:cholesterol was found effective in eliminating biofilm-producing bacteria^115^. Thus, the property of liposome-encapsulated drugs to overcome AMR due to impermeable OM, efflux mechanisms, enzymatic degradation, and biofilm formation make it an ideal strategy to restore treatment options against clinically important MDR bacterial pathogens. However, specificity of liposomes to target bacterial cells only needs to be optimized^115^. To this end, *P. aeruginosa* targeted liposomes with a glycomimetic-decorated surface have recently been reported^116^.

#### Dendrimers

Dendrimers (Ds) have emerged as an innovative approach targeting MDR Gram-negative pathogens. Generally, dendrimers are branched macromolecules made up of a surface functional group, a core unit, and a branching unit attached to a core. Different families of Ds with different mechanism of action have been studied including glycodendrimers (resist toxin binding to eukaryotic cells, interfere with bacterial adhesion), cationic dendrimers (disrupts bacterial membranes), anionic dendrimers (detergent-like action) and peptidic dendrimers (disrupt bacterial membrane or interact with their intracellular components)^117^. Cationic Ds as drug delivery system play a significant role in the field of nanomedicine. Some cationic Ds show synergy with commonly used antibiotics and are found to have antibiofilm effects or antimicrobial effects against MDR bacteria^118^. Cationic Ds such as polyamidoamine (PAMAM) Ds and propylene imine (PPI) Ds carry a large number of peripheral ionizable primary and tertiary amine groups, which give them a high positive charge and correspondingly high antibacterial activity. Most representative cationic PAMAM Ds and PPI Ds developed include levofloxacin (0.1 μg/mL) co-administered with highly maltose-coated 3GPPIs (PPI-G3-DS-Mal), C16-DABCO-loaded mannose-terminated 4G PAMAM Ds, G7 PAMAM-D, self-assembly poly (aryl ether)-PAMAM-based amphiphilic Ds, CdS/Ag2S (QDs)-loaded PAMAM Ds/MWCNTs, G1-G4 NO-releasing-alkyl(QA)-PAMAM Ds, 1G NO-releasing octyl- and dodecyl-modified PAMAM Ds^119^. Besides, the synergistic effect of PPI glycodendrimers with a dense maltose shell (PPI-G3-DS-Mal) with levofloxacin is reported to reduce the growth of *E. coli* and the same dendrimer when combined with amoxicillin shows a synergistic effect against Gram-negative bacteria by potentiating the antimicrobial activity of amoxicillin^118^.

Schito and colleagues investigated the antimicrobial activity of three non-toxic, 5th generation polyester-based cationic dendrimers G5Ds:G5H, G5K, and G5HK against several MDR clinical strains. All the dendrimers were found to potentially target MDR non-fermenting GNB *P. aeruginosa*, *S. maltophilia,* and *A. baumannii* with MICs from 0.5 to 33 µM. Time kill and turbidimetric studies of G5K revealed a rapid, non-lytic bactericidal activity. Additionally, their non-cytotoxic nature makes them an attractive target to be investigated further for their drug-like properties^119^. Reymond et al. investigated the antimicrobial efficacy of a peptidic dendrimer, G3KL. The structure of the molecule contains leucine and branched chain lysine residues. Activity of G3KL was tested against 32 strains of *A. baumannii* and 35 strains of *P. aeruginosa*. G3KL displayed a MIC of 8 µg/mL against all tested strains^120^. However, its large size and issues in its synthesis were the matter of concern. Therefore, the authors designed a new antimicrobial peptide dendrimer G2KL by reducing the number of amino acids. But the modification diminished its antimicrobial efficacy against *E. coli, P. aeruginosa,* and *Bacillus subtilis*. Considering this diminished activity, they modified G2KL by adding a short fatty acid chain, formulating TNS18, which displayed good activity against MDR strains of *A. baumannii, P. aeruginosa*, and *E. coli* with a MIC of 4–8 µg/ml^121^. Despite excellent antibacterial efficacy, dendrimers call for careful optimization for their low biodegradability, susceptibility to opsonization, toxicity to mammalian cells, including hemolytic toxicity, cytotoxicity, and hematological toxicity, as well as fast clearance^117^.

#### Fragment-based drug discovery (FBDD)

Another innovative approach of developing a drug molecule is based on fragment-based drug design. This strategy involves finding a new drug/scaffold based on the screening of compound libraries with MW < 300 g/mol. FBDD utilizes strategies such as differential scanning fluorimetry, isothermal titration microcalorimetry, nuclear magnetic resonance, surface plasmon resonance, and X-ray crystallography. Binders are selected for their further synthetic remodeling into drug molecules by fragment growing or fragment merging. This FBDD process gains attention in the field of target-based drug discovery because of its interesting advantages such as minimal experimental costs and offering diverse hits^122^. Moreau and colleagues screened ~25,000 fragments from Novartis fourth-generation core fragment library targeting the bacterial phosphopantetheine-adenylyl-transferase (PPAT). The enzyme is involved in CoA biosynthesis, but interestingly, does not show high sequence homology with its human analog and thus can be an attractive target of drug molecules. From 25,000 fragments, 631 hits showing good binding affinities with the target (i.e., displaying ≥30% inhibition) were studied further. Triazolopyrimidinone and azabenzimidazole were identified to bind bacterial PPAT. However, the antibacterial activity was against efflux pump-deficient strains^123^. Similarly, Mansbach et al. screened a fragment library in silico to target OM of Gram-negative bacteria and 43 fragments were predicted to cross the OM. These fragments were then experimentally studied for their OM permeabilizing efficacy and the eight compounds depicting highest OM permeability based on a computational algorithm were studied for their antibacterial activity against *P. aeruginosa* and *A. baumannii*, of which five compounds displayed potent activity (µP∆6Pore/µP∆6 > 0.8) against *P. aeruginosa*^124^.

## Conclusion

Gram-negative bacteria are amongst the most difficult-to-treat bacteria. This primarily results from their uniquely complex cell envelope, which not only acts as a barrier for the entry of antibiotics into the cell but also depletes accumulating drugs by efflux pumps. New antibiotics targeting intracellular processes are being developed, but transporting these drugs across the highly impermeable OM is the major hurdle. Antibiotics targeting the OM, such as outer membrane permeabilizers, OM protein targeting compounds, and compounds entering through porins are currently available.

However, MDR Gram-negative bacteria have evolved resistance mechanisms to escape from the action of these drugs by mutations or the action of drug-targeting enzymes. Unfortunately, resistance against drugs currently in clinical trials is also already observed. Taken together, the emergence of AMR in drugs is inevitable and multiple resistances can accumulate in single pathogens. Therefore, we must prepare for the threat of total drug resistance^21^.

Physicochemical parameters of antimicrobials have been shown to play an important role for accumulation into Gram-negative bacteria. Correlating these requires both high-throughput and structure-agnostic accumulation assays^38^. This review highlights the recent ways of targeting the highly selective OM of Gram-negative bacteria by tuning physicochemical properties of drugs or other innovative approaches to overcome the OM. Since we are in the antimicrobial resistance era, we must take further action to develop more effective antibiotics with strong resistance-defeating capabilities.

### Reporting summary

Further information on research design is available in the Nature Research Reporting Summary linked to this article.

## Supplementary information

Reporting Summary

## References

[1] Bassetti, M. & Garau, J. Current and future perspectives in the treatment of multidrug-resistant Gram-negative infections. *J Antimicrob. Chemother.* **76**, iv23–iv37. 10.1093/jac/dkab352 (2021). 34849997 10.1093/jac/dkab352 PMC8632738

[2] Antimicrobial Resistance Collaborators. Global burden of bacterial antimicrobial resistance in 2019: a systematic analysis. *Lancet* **399**, 629–655 (2022). 35065702 10.1016/S0140-6736(21)02724-0 PMC8841637

[3] O’Neill, J. Antimicrobial resistance: tackling a crisis for the health and wealth of nations. Rev. Antimicrob. Resist. Grande-Bretagne https://amrreview.org/sites/default/files/AMR%20Review%20Paper%20%20Tackling%20a%20crisis%20for%20the%20health%20and%20wealth%20of%20nations_1.pdf (2014).

[4] Miethke, M. et al. Towards the sustainable discovery and development of new antibiotics. *Nat. Rev. Chem.* **5**, 726–749 (2021). 34426795 10.1038/s41570-021-00313-1 PMC8374425

[5] Cochrane, S. A. et al. Antimicrobial lipopeptide tridecaptin A1 selectively binds to Gram-negative lipid II. *Proc. Natl Acad. Sci. USA* **113**, 11561–11566 (2016). 27688760 10.1073/pnas.1608623113 PMC5068289

[6] Delcour, A. H. Outer membrane permeability and antibiotic resistance. *Biochim. Biophys. Acta* **1794**, 808–816 (2009). 19100346 10.1016/j.bbapap.2008.11.005 PMC2696358

[7] Boucher, H. W. Bad bugs, no drugs 2002-2020: progress, challenges, and call to action. *Trans. Am. Clin. Climatol. Assoc.* **131**, 65–71 (2020). 32675844 PMC7358500

[8] Choi, U. & Lee, C. R. Distinct roles of outer membrane porins in antibiotic resistance and membrane integrity in *Escherichia coli*. *Front. Microbiol.* **10**, 953 (2019). 31114568 10.3389/fmicb.2019.00953 PMC6503746

[9] Wesseling, C. M. J. & Martin, N. I. Synergy by perturbing the Gram-Negative outer membrane: opening the door for Gram-positive specific antibiotics. *ACS Infect. Dis.* **8**, 1731–1757 (2022). 35946799 10.1021/acsinfecdis.2c00193 PMC9469101

[10] Raetz, C. R. H., Reynolds, C. M., Trent, M. S. & Bishop, R. E. Lipid a modification systems in Gram-negative bacteria. *Annu. Rev. Biochem.* **76**, 295–329 (2007). 17362200 10.1146/annurev.biochem.76.010307.145803 PMC2569861

[11] Tiz, D. B., Kikelj, D. & Zidar, N. Expert opinion on drug discovery overcoming problems of poor drug penetration into bacteria: challenges and strategies for medicinal chemists. *Expert Opin. Drug Discov.* **00**, 1–11 (2018). 10.1080/17460441.2018.1455660 29566560

[12] Amaral, L., Martins, A., Spengler, G. & Molnar, J. Efflux pumps of Gram-negative bacteria: what they do, how they do it, with what and how to deal with them. *Front. Pharmacol.* **4**, 168 (2014). 24427138 10.3389/fphar.2013.00168 PMC3879458

[13] McMurry, L., Petrucci, R. E. J. & Levy, S. B. Active efflux of tetracycline encoded by four genetically different tetracycline resistance determinants in *Escherichia coli*. *Proc. Natl Acad. Sci. USA* **77**, 3974–3977 (1980). 7001450 10.1073/pnas.77.7.3974 PMC349750

[14] Alegun, O. Mechanism of antibiotic permeability through the Gram-negative bacterial envelope https://uknowledge.uky.edu/chemistry_etds/156/.

[15] Ramirez, M. S. & Tolmasky, M. E. Aminoglycoside modifying enzymes. *Drug Resist. Updates* **13**, 151–171 (2010). 10.1016/j.drup.2010.08.003 PMC2992599 20833577

[16] Egorov, A. M., Ulyashova, M. M. & Rubtsova, M. Y. Bacterial enzymes and antibiotic resistance. *Acta Nat.* **10**, 33–48 (2018). PMC6351036 30713760

[17] Darby, E. M. et al. Molecular mechanisms of antibiotic resistance revisited. *Nat. Rev. Microbiol.* **21**, 280–295 (2023). 36411397 10.1038/s41579-022-00820-y

[18] MacNair, C. R. & Tan, M. W. The role of bacterial membrane vesicles in antibiotic resistance. *Ann. N.Y. Acad. Sci.* **1519**, 63–73 (2023). 36415037 10.1111/nyas.14932

[19] Silver, L. L. A Gestalt approach to Gram-negative entry. *Bioorg. Med. Chem.* **24**, 6379–6389 (2016). 27381365 10.1016/j.bmc.2016.06.044

[20] Cama, J., Henney, A. M. & Winterhalter, M. Breaching the barrier: quantifying antibiotic permeability across gram-negative bacterial membranes. *J. Mol. Biol.* **431**, 3531–3546 (2019). 30959052 10.1016/j.jmb.2019.03.031

[21] Payne, D. J., Gwynn, M. N., Holmes, D. J. & Pompliano, D. L. Drugs for bad bugs: confronting the challenges of antibacterial discovery. *Nat. Rev. Drug Discov.* **6**, 29–40 (2007). 17159923 10.1038/nrd2201

[22] Vaara, M. Agents that increase the permeability of the outer membrane. *Microbiol. Rev.* **56**, 395–411 (1992). 1406489 10.1128/mr.56.3.395-411.1992 PMC372877

[23] Alcaraz, A., Nestorovich, E. M., Aguilella-Arzo, M., Aguilella, V. M. & Bezrukov, S. M. Salting out the ionic selectivity of a wide channel: the asymmetry of OmpF. *Biophys. J.* **87**, 943–957 (2004). 15298901 10.1529/biophysj.104/043414 PMC1304502

[24] Cohen, S. P. et al. Endogenous active efflux of norfloxacin in susceptible *Escherichia coli*. *Antimicrob. Agents Chemother.* **32**, 1187–1191 (1988). 3056253 10.1128/aac.32.8.1187 PMC172374

[25] Chevalier, J., Malléa, M. & Pagès, J. M. Comparative aspects of the diffusion of norfloxacin, cefepime and spermine through the F porin channel of *Enterobacter cloacae*. *Biochem. J.* **348**, 223–227 (2000). 10794735 PMC1221057

[26] Charrel, R. N., Pages, J. M., De Micco, P. & Mallea, M. Prevalence of outer membrane porin alteration in beta-lactam-antibiotic-resistant Enterobacter aerogenes. *Antimicrob. Agents Chemother.* **40**, 2854–2858 (1996). 9124854 10.1128/aac.40.12.2854 PMC163635

[27] Chopra, I. Molecular mechanisms involved in the transport of antibiotics into bacteria. *Parasitology* **96**, S25–S44 (1988). 3287290 10.1017/s0031182000085966

[28] McCreary, E. K., Heil, E. L. & Tamma, P. D. New perspectives on antimicrobial agents: cefiderocol. *Antimicrob. Agents Chemother.* **65**, e0217120 (2021). 34031052 10.1128/AAC.02171-20 PMC8373209

[29] Kaye, K. S. et al. Fosfomycin for injection (ZTI-01) versus piperacillin-tazobactam for the treatment of complicated urinary tract infection including acute pyelonephritis: ZEUS, a phase 2/3 randomized trial. *Clin. Infect. Dis.* **69**, 2045–2056 (2019). 30861061 10.1093/cid/ciz181 PMC6880332

[30] Avent, M. L. et al. Fosfomycin: what was old is new again. *Intern. Med. J.* **48**, 1425–1429 (2018). 30517987 10.1111/imj.14122

[31] Hawkey, P. M. et al. Treatment of infections caused by multidrug-resistant Gram-negative bacteria: report of the British Society for Antimicrobial Chemotherapy/Healthcare Infection Society/British Infection Association Joint Working Party. *J. Antimicrob. Chemother.* **73**, iii2–iii78 (2018). 29514274 10.1093/jac/dky027

[32] US Food and Drug Administration. Tygacil (tigecycline): prescribing information. https://www.accessdata.fda.gov/drugsatfda_docs/label/2020/021821s049lbl.pdf.

[33] Chastain, D. B., White, B. P., Cretella, D. A. & Bland, C. M. Is it time to rethink the notion of carbapenem-sparing therapy against extended-spectrum β-lactamase-producing Enterobacteriaceae bloodstream infections? A critical review. *Ann. Pharmacother.* **52**, 484–492 (2018). 29239220 10.1177/1060028017748943

[34] Iskandar, K. et al. Antibiotic discovery and resistance: the chase and the race. *Antibiotics* **11**, 1–38 (2022). 10.3390/antibiotics11020182 PMC8868473 35203785

[35] Leusmann, S., Ménová, P., Shanin, E., Titz, A. & Rademacher, C. Glycomimetics for the inhibition and modulation of lectins.*Chem. Soc. Rev.* **52**, 3663–3740 (2023). https://pubs.rsc.org/en/content/articlelanding/2023/cs/d2cs00954d. 37232696 10.1039/d2cs00954d PMC10243309

[36] Lakemeyer, M., Zhao, W., Mandl, F. A., Hammann, P. & Sieber, S. A. Thinking outside the box-novel antibacterials to tackle the resistance crisis. *Angew. Chem. (Int. Ed. Engl.)* **57**, 14440–14475 (2018). 29939462 10.1002/anie.201804971

[37] Mohapatra, S. S., Dwibedy, S. K. & Padhy, I. Polymyxins, the last-resort antibiotics: mode of action, resistance emergence, and potential solutions. *J. Biosci.* **46**, 85 (2021). 34475315 10.1007/s12038-021-00209-8 PMC8387214

[38] Zhao, S. et al. Defining new chemical space for drug penetration into Gram-negative bacteria. *Nat. Chem. Biol.* **16**, 1293–1302 (2020). 33199906 10.1038/s41589-020-00674-6 PMC7897441

[39] Lipinski, C. A., Lombardo, F., Dominy, B. W. & Feeney, P. J. Experimental and computational approaches to estimate solubility and permeability in drug discovery and development settings. *Adv. Drug Deliv. Rev.* **46**, 3–26 (2001). 11259830 10.1016/s0169-409x(00)00129-0

[40] O’Shea, R. & Moser, H. E. Physicochemical properties of antibacterial compounds: implications for drug discovery. *J. Med. Chem.* **51**, 2871–2878 (2008). 18260614 10.1021/jm700967e

[41] Muñoz, K. A. & Hergenrother, P. J. Facilitating compound entry as a means to discover antibiotics for Gram-negative bacteria. *Acc. Chem. Res.* **54**, 1322–1333 (2021). 33635073 10.1021/acs.accounts.0c00895 PMC7969460

[42] Scott, L. J. Delafloxacin: a review in acute bacterial skin and skin structure infections. *Drugs* **80**, 1247–1258 (2020). 32666425 10.1007/s40265-020-01358-0 PMC7497496

[43] Intravenous Pentamidine for Pneumocystis jirovecii Pneumonia https://clinicaltrials.gov/ct2/show/NCT02669706?term=pentamidine&draw=2&rank=1.

[44] Brown, K. S., Reed, M. D., Dalal, J. & Makii, M. D. Tolerability of aerosolized versus intravenous pentamidine for *Pneumocystis jirovecii* pneumonia prophylaxis in immunosuppressed pediatric, adolescent, and young adult patients. *J. Pediatr. Pharmacol. Ther.* **25**, 111–116 (2020). 32071585 10.5863/1551-6776-25.2.111 PMC7025749

[45] Dorlo, T. P. C. & Kager, P. A. Pentamidine dosage: a base/salt confusion. *PLoS Negl. Trop. Dis.* **2**, e225 (2008). 18509543 10.1371/journal.pntd.0000225 PMC2387188

[46] Libman, M. D., Miller, M. A. & Richards, G. K. Antistaphylococcal activity of pentamidine. *Antimicrob. Agents Chemother.* **34**, 1795–1796 (1990). 2285292 10.1128/aac.34.9.1795 PMC171928

[47] Maciejewska, D. et al. In vitro screening of pentamidine analogs against bacterial and fungal strains. *Bioorg. Med. Chem. Lett.* **24**, 2918–2923 (2014). 24830598 10.1016/j.bmcl.2014.04.075

[48] Stokes, J. M. et al. Pentamidine sensitizes Gram-negative pathogens to antibiotics and overcomes acquired colistin resistance. *Nat. Microbiol.* **2**, 17028 (2017). 28263303 10.1038/nmicrobiol.2017.28 PMC5360458

[49] Amos, H. & Vollmayer, E. Effect of pentamidine on the growth of Escherichia coli. *J. Bacteriol.* **73**, 172–177 (1957). 13416166 10.1128/jb.73.2.172-177.1957 PMC289771

[50] Wesseling, C. M. J., Slingerland, C. J., Veraar, S., Lok, S. & Martin, N. I. Structure-activity studies with bis-amidines that potentiate Gram-positive specific antibiotics against Gram-negative pathogens. *ACS Infect. Dis.* **7**, 3314–3335 (2021). 34766746 10.1021/acsinfecdis.1c00466 PMC8669655

[51] Zhao, R. et al. Effectiveness of ertapenem for treatment of infections in children: an evidence mapping and meta-analysis. *Front. Pediatr.* **10**, 982179 (2022). 36324821 10.3389/fped.2022.982179 PMC9620802

[52] Counter, F. T. et al. Synthesis and antimicrobial evaluation of dirithromycin (AS-E 136; LY237216), a new macrolide antibiotic derived from erythromycin. *Antimicrob. Agents Chemother.* **35**, 1116–1126 (1991). 1929252 10.1128/aac.35.6.1116 PMC284297

[53] Massey, E. H., Kitchell, B. S., Martin, L. D. & Gerzon, K. Antibacterial activity of 9(S)-erythromycylamine-aldehyde condensation products. *J. Med. Chem.* **17**, 105–107 (1974). 4585970 10.1021/jm00247a018

[54] Farmer, S., Li, Z. S. & Hancock, R. E. Influence of outer membrane mutations on susceptibility of *Escherichia coli* to the dibasic macrolide azithromycin. *J. Antimicrob. Chemother.* **29**, 27–33 (1992). 1310670 10.1093/jac/29.1.27

[55] Ropponen, H. K. et al. Assessment of the rules related to gaining activity against Gram-negative bacteria. *RSC Med. Chem.* **12**, 593–601 (2021). 34046630 10.1039/d0md00409j PMC8128065

[56] Macnair, C. R., Tsai, C. N. & Brown, E. D. Creative targeting of the Gram-negative outer membrane in antibiotic discovery. *Ann. N.Y. Acad. Sci.* **1459**, 69–85 (2020). 31762048 10.1111/nyas.14280

[57] Jenkins, R. J. & Dotson, G. D. Dual targeting antibacterial peptide inhibitor of early lipid A biosynthesis. *ACS Chem. Biol.* **7**, 1170–1177 (2012). 22530734 10.1021/cb300094a PMC3401278

[58] García-Quintanilla, M. et al. Inhibition of LpxC Increases antibiotic susceptibility in *Acinetobacter baumannii*. *Antimicrob. Agents Chemother.* **60**, 5076–5079 (2016). 27270288 10.1128/AAC.00407-16 PMC4958213

[59] Vinuesa, V., Cruces, R., Nonnoi, F. & McConnell, M. J. Inhibition of LpxC increases the activity of iron chelators and gallium nitrate in multidrug-resistant *Acinetobacter baumannii*. *Antibiotics* **10**, 609 (2011). 10.3390/antibiotics10050609 PMC8160660 34065605

[60] Imai, Y. et al. A new antibiotic selectively kills Gram-negative pathogens. *Nature* **576**, 459–464 (2019). 31747680 10.1038/s41586-019-1791-1 PMC7188312

[61] Seyfert, C. E. et al. Darobactins exhibiting superior antibiotic activity by cryo-em structure-guided biosynthetic engineering. *Angew. Chem. (Int. Ed. Engl.).* **62**, e202214094 (2023). 36308277 10.1002/anie.202214094 PMC10107326

[62] Bohnert, J. A. & Kern, W. V. Selected arylpiperazines are capable of reversing multidrug resistance in Escherichia coli overexpressing RND efflux pumps. *Antimicrob. Agents Chemother.* **49**, 849–852 (2015). 10.1128/AAC.49.2.849-852.2005 PMC547223 15673787

[63] Yoshida, K.-I. et al. MexAB-OprM specific efflux pump inhibitors in *Pseudomonas aeruginosa*. Part 7: highly soluble and in vivo active quaternary ammonium analogue D13-9001, a potential preclinical candidate. *Bioorg. Med. Chem.* **15**, 7087–7097 (2007). 17869116 10.1016/j.bmc.2007.07.039

[64] Otręebska-Machaj, E. et al. Efflux pump blockers in Gram-negative bacteria: the new generation of hydantoin based-modulators to improve antibiotic activity. *Front. Microbiol.* **7**, 622 (2016). 27199950 10.3389/fmicb.2016.00622 PMC4853399

[65] Benedetto Tiz, D., Kikelj, D. & Zidar, N. Overcoming problems of poor drug penetration into bacteria: challenges and strategies for medicinal chemists. *Expert Opin. Drug Discov.* **13**, 497–507 (2018). 29566560 10.1080/17460441.2018.1455660

[66] AlMatar, M., Albarri, O., Makky, E. A. & Köksal, F. Efflux pump inhibitors: new updates. *Pharmacol. Rep.* **73**, 1–16 (2021). 32946075 10.1007/s43440-020-00160-9

[67] Odds, F. C. Synergy, antagonism, and what the chequerboard puts between them. *J. Antimicrob. Chemother.* **52**, 1 (2003). 12805255 10.1093/jac/dkg301

[68] Zavascki, A. P. & Nation, R. L. Nephrotoxicity of polymyxins: is there any difference between colistimethate and polymyxin B? *Antimicrob. Agents Chemother.* **61**, e02319–16 (2017). 27993859 10.1128/AAC.02319-16 PMC5328560

[69] Olaitan, A. O., Morand, S. & Rolain, J. M. Mechanisms of polymyxin resistance: acquired and intrinsic resistance in bacteria. *Front. Microbiol.* **5**, 643 (2014). 25505462 10.3389/fmicb.2014.00643 PMC4244539

[70] French, S. et al. Potentiation of antibiotics against Gram-negative bacteria by polymyxin B analogue SPR741 from unique perturbation of the outer membrane. *ACS Infect. Dis.* **6**, 1405–1412 (2020). 31566948 10.1021/acsinfecdis.9b00159

[71] MacNair, C. R. et al. Overcoming mcr-1 mediated colistin resistance with colistin in combination with other antibiotics. *Nat. Commun.* **9**, 458 (2018). 29386620 10.1038/s41467-018-02875-z PMC5792607

[72] Tyrrell, J. M., Aboklaish, A. F., Walsh, T. R., Vaara, T. & Vaara, M. The polymyxin derivative NAB739 is synergistic with several antibiotics against polymyxin-resistant strains of *Escherichia coli, Klebsiella pneumoniae* and *Acinetobacter baumannii*. *Peptides* **112**, 149–153 (2019). 30586602 10.1016/j.peptides.2018.12.006

[73] Klobucar, K. et al. Genetic and chemical screening reveals targets and compounds to potentiate gram-positive antibiotics against gram-negative bacteria. *ACS Infect. Dis.* **8**, 2187–2197 (2022). 36098580 10.1021/acsinfecdis.2c00357

[74] Moon, S. H. et al. Novel linear lipopeptide paenipeptins with potential for eradicating biofilms and sensitizing Gram-negative bacteria to rifampicin and clarithromycin. *J. Med. Chem.* **60**, 9630–9640 (2017). 29136469 10.1021/acs.jmedchem.7b01064 PMC12124638

[75] Domalaon, R. et al. Dilipid ultrashort cationic lipopeptides as adjuvants for chloramphenicol and other conventional antibiotics against Gram-negative bacteria. *Amino Acids* **51**, 383–393 (2019). 30392097 10.1007/s00726-018-2673-9

[76] Schweizer, L., Ramirez, D. & Schweizer, F. Effects of lysine *N*-ζ-methylation in ultrashort tetrabasic lipopeptides (UTBLPs) on the potentiation of rifampicin, novobiocin, and niclosamide in Gram-negative bacteria. *Antibiotics* **11**, 335 (2022). 35326798 10.3390/antibiotics11030335 PMC8963254

[77] Domalaon, R., Idowu, T., Zhanel, G. G. & Schweizer, F. Antibiotic hybrids: the next generation of agents and adjuvants against Gram-negative pathogens? *Clin Microbiol. Rev.* **31**, e00077–17 (2018). 29540434 10.1128/CMR.00077-17 PMC5967690

[78] Gorityala, B. K. et al. Hybrid antibiotic overcomes resistance in *P. aeruginosa* by enhancing outer membrane penetration and reducing efflux. *J. Med. Chem.* **59**, 8441–8455 (2016). 27524179 10.1021/acs.jmedchem.6b00867

[79] Gorityala, B. K. et al. Adjuvants based on hybrid antibiotics overcome resistance in *Pseudomonas aeruginosa* and enhance fluoroquinolone efficacy. *Angew. Chem. (Int. Ed. in Engl.)* **55**, 555–559 (2016). 26610184 10.1002/anie.201508330

[80] Lyu, Y. et al. Amphiphilic tobramycin-lysine conjugates sensitize multidrug resistant Gram-negative bacteria to rifampicin and minocycline. *J. Med. Chem.* **60**, 3684–3702 (2017). 28409644 10.1021/acs.jmedchem.6b01742

[81] Ghosh, C. et al. Small molecular antibacterial peptoid mimics: the simpler the better! *J. Med. Chem.* **57**, 1428–1436 (2014). 24479371 10.1021/jm401680a

[82] Idowu, T., Ammeter, D., Rossong, H., Zhanel, G. G. & Schweizer, F. Homodimeric tobramycin adjuvant repurposes novobiocin as an effective antibacterial agent against Gram-negative bacteria. *J. Med. Chem.* **62**, 9103–9115 (2019). 31557020 10.1021/acs.jmedchem.9b00876

[83] Yang, X., Domalaon, R., Lyu, Y., Zhanel, G. G. & Schweizer, F. Tobramycin-linked efflux pump inhibitor conjugates synergize fluoroquinolones, rifampicin and fosfomycin against multidrug-resistant *Pseudomonas aeruginosa*. *J. Clin. Med.* **7**, 158 (2018). 29932132 10.3390/jcm7070158 PMC6069439

[84] Berry, L., Domalaon, R., Brizuela, M., Zhanel, G. G. & Schweizer, F. Polybasic peptide-levofloxacin conjugates potentiate fluoroquinolones and other classes of antibiotics against multidrug-resistant Gram-negative bacteria. *MedChemComm* **10**, 517–527 (2019). 31057731 10.1039/c9md00051h PMC6482413

[85] Meiers, J., Rox, K. & Titz, A. Lectin-targeted prodrugs activated by *Pseudomonas aeruginosa* for self-destructive antibiotic release. *J Med. Chem.* **65**, 13988–14014 (2022). 36201248 10.1021/acs.jmedchem.2c01214 PMC9619409

[86] Meiers, J. et al. Directing drugs to bugs: antibiotic-carbohydrate conjugates targeting biofilm-associated lectins of *Pseudomonas aeruginosa*. *J. Med. Chem.* **63**, 11707–11724 (2020). 32924479 10.1021/acs.jmedchem.0c00856 PMC7586336

[87] O’Callaghan, C. H., Sykes, R. B. & Staniforth, S. E. A new cephalosporin with a dual mode of action. *Antimicrob. Agents Chemother.* **10**, 245–248 (1976). 984765 10.1128/aac.10.2.245 PMC429729

[88] Mobashery, S. & Johnston, M. Inactivation of alanine racemase by beta-chloro-L-alanine released enzymatically from amino acid and peptide C10-esters of deacetylcephalothin. *Biochemistry.* **26**, 5878–5884 (1987). 3118951 10.1021/bi00392a045

[89] Mobashery, S., Lerner, S. A. & Johnston, M. Conscripting .beta.-lactamase for use in drug delivery. Synthesis and biological activity of a cephalosporin C10-ester of an antibiotic dipeptide. *J. Am. Chem. Soc.* **108**, 1685–1686 (1986).

[90] Li, Qing et al. NB2001, a novel antibacterial agent with broad-spectrum activity and enhanced potency against beta-lactamase-producing strains. *Antimicrob. Agents Chemother.* **46**, 1262–1268 (2002). 11959554 10.1128/AAC.46.5.1262-1268.2002 PMC127132

[91] Jones, R. N., Barry, A. L. & Thornsberry, C. Antimicrobial activity of Ro 23-9424, a novel ester-linked codrug of fleroxacin and desacetylcefotaxime. *Antimicrob. Agents Chemother.* **33**, 944–950 (1989). 2504106 10.1128/aac.33.6.944 PMC284260

[92] Russell, A. D. Effect of magnesium ions and ethylenediamine tetra-acetic acid on the activity of vancomycin against *Escherichia coli* and *Staphylococcus aureus*. *J. Appl. Bacteriol.* **30**, 395–401 (1967). 4965331 10.1111/j.1365-2672.1967.tb00314.x

[93] Ayres, H. M., Furr, J. R. & Russell, A. D. Effect of permeabilizers on antibiotic sensitivity of *Pseudomonas aeruginosa*. *Lett. Appl. Microbiol.* **28**, 13–16 (1999). 10030025 10.1046/j.1365-2672.1999.00486.x

[94] Hemaiswarya, S. & Doble, M. Ã. Synergistic interaction of eugenol with antibiotics against Gram-negative bacteria. *Phytomedicine.* **16**, 997–1005 (2009). 19540744 10.1016/j.phymed.2009.04.006

[95] Faraldo-Gómez, J. D. & Sansom, M. S. P. Acquisition of siderophores in Gram-negative bacteria. *Nat. Rev. Mol. Cell Biol.* **4**, 105–116 (2003). 12563288 10.1038/nrm1015

[96] Mislin, G. L. A. & Schalk, I. J. Siderophore-dependent iron uptake systems as gates for antibiotic Trojan horse strategies against *Pseudomonas aeruginosa*. *Metall. Integr. Biometal Sci.* **6**, 408–420 (2014). 10.1039/c3mt00359k 24481292

[97] de Carvalho, C. C. C. R. & Fernandes, P. Siderophores as “Trojan Horses”: tackling multidrug resistance? *Front. Microbiol.* **5**, 290 (2014). 24971080 10.3389/fmicb.2014.00290 PMC4053685

[98] Al Shaer, D., Al Musaimi, O., de la Torre, B. G. & Albericio, F. Hydroxamate siderophores: natural occurrence, chemical synthesis, iron binding affinity and use as Trojan horses against pathogens. *Eur. J. Med. Chem.* **208**, 112791 (2020). 32947228 10.1016/j.ejmech.2020.112791

[99] Ji, C., Miller, P. A. & Miller, M. J. Iron transport-mediated drug delivery: practical syntheses and in vitro antibacterial studies of tris-catecholate siderophore-aminopenicillin conjugates reveals selectively potent antipseudomonal activity. *J. Am. Chem. Soc.* **134**, 9898–9901 (2012). 22656303 10.1021/ja303446w PMC3380153

[100] Zhanel, G. G. et al. Cefiderocol: a siderophore cephalosporin with activity against carbapenem-resistant and multidrug-resistant Gram-Negative Bacilli. *Drugs* **79**, 271–289 (2019). 30712199 10.1007/s40265-019-1055-2

[101] Pinkert, L. et al. Antibiotic conjugates with an artificial MECAM-based siderophore are potent agents against Gram-Positive and Gram-Negative bacterial pathogens. *J. Med. Chem.* **64**, 15440–15460 (2021). 34619959 10.1021/acs.jmedchem.1c01482

[102] Karakonstantis, S., Rousaki, M. & Kritsotakis, E. I. Cefiderocol: systematic review of mechanisms of resistance, heteroresistance and in vivo emergence of resistance. *Antibiotics* **11**, 723 (2022). 35740130 10.3390/antibiotics11060723 PMC9220290

[103] Jousset, A. B. et al. Rapid selection of a cefiderocol-resistant *Escherichia coli* producing NDM-5 associated with a single amino acid substitution in the CirA siderophore receptor. *J. Antimicrob. Chemother.* **78**, 1125–1127 (2023). 36864015 10.1093/jac/dkad004

[104] Wang, Q. et al. occurrence of high levels of cefiderocol resistance in carbapenem-resistant *Escherichia coli* before its approval in China: a report from China CRE-Network. *Microbiol. Spectr.* **10**, e0267021 (2022). 35481835 10.1128/spectrum.02670-21 PMC9241927

[105] Cascales, E. et al. Colicin biology. *Microbiol. Mol. Biol. Rev.* **71**, 158–229 (2007). 17347522 10.1128/MMBR.00036-06 PMC1847374

[106] Biswas, K., Upadhayay, S., Rapsang, G. F. & Joshi, S. R. Antibacterial and synergistic activity against β-lactamase-producing nosocomial bacteria by bacteriocin of LAB isolated from lesser known traditionally fermented products of India. *HAYATI J. Biosci.* **24**, 87–95 (2017).

[107] Ghequire, M. G. K., Swings, T., Michiels, J., Buchanan, S. K. & De Mot, R. Hitting with a BAM: selective killing by lectin-like bacteriocins. *MBio.* **9**, e02138–17 (2018). 29559575 10.1128/mBio.02138-17 PMC5874912

[108] Gradisteanu Pircalabioru, G. et al. Bacteriocins in the era of antibiotic resistance: Rising to the challenge. *Pharmaceutics* **13**, 196 (2021). 33540560 10.3390/pharmaceutics13020196 PMC7912925

[109] Malanovic, N., Ön, A., Pabst, G., Zellner, A. & Lohner, K. Octenidine: Novel insights into the detailed killing mechanism of Gram-negative bacteria at a cellular and molecular level. *Int. J. Antimicrob. Agents* **56**, 106146 (2020). 32853670 10.1016/j.ijantimicag.2020.106146

[110] Perez Sirkin, Y. A., Tagliazucchi, M. & Szleifer, I. Transport in nanopores and nanochannels: some fundamental challenges and nature-inspired solutions. *Mater. Today Adv.* **5**, 100047 (2020).

[111] Schirmer, T. & Phale, P. S. Brownian dynamics simulation of ion flow through porin channels. *J. Mol. Biol.* **294**, 1159–1167 (1999). 10600374 10.1006/jmbi.1999.3326

[112] Wang, J., Prajapati, J. D., Kleinekathöfer, U. & Winterhalter, M. Dynamic interaction of fluoroquinolones with magnesium ions monitored using bacterial outer membrane nanopores. *Chem. Sci.* **11**, 10344–10353 (2020). 34094296 10.1039/d0sc03486j PMC8162440

[113] Mugabe, C., Halwani, M., Azghani, A. O., Lafrenie, R. M. & Omri, A. Mechanism of enhanced activity of liposome-entrapped aminoglycosides against resistant strains of Pseudomonas aeruginosa. *Antimicrob. Agents Chemother.* **50**, 2016–2022 (2006). 16723560 10.1128/AAC.01547-05 PMC1479138

[114] Nacucchio, M. C., Bellora, M. J., Sordelli, D. O. & D’Aquino, M. Enhanced liposome-mediated activity of piperacillin against staphylococci. *Antimicrob. Agents Chemother.* **27**, 137–139 (1985). 3872624 10.1128/aac.27.1.137 PMC176221

[115] Ferreira, M. et al. Liposomes as antibiotic delivery systems: a promising nanotechnological strategy against antimicrobial resistance. *Molecules* **26**, 2047 (2021). 33918529 10.3390/molecules26072047 PMC8038399

[116] Metelkina, O. et al. Targeting extracellular lectins of *Pseudomonas aeruginosa* with glycomimetic liposomes. *J. Mater. Chem. B* **10**, 537–548 (2022). 34985094 10.1039/d1tb02086b

[117] Alfei, S., & Schito, A. M. From nanobiotechnology, positively charged biomimetic dendrimers as novel antibacterial agents: a review. *Nanomaterials* **10**10.3390/nano10102022 (2020). 10.3390/nano10102022 PMC7602242 33066468

[118] Wrońska, N., Majoral, J. P., Appelhans, D., Bryszewska, M. & Lisowska, K. Synergistic effects of anionic/cationic dendrimers and levofloxacin on antibacterial activities. *Molecules.* **24**, 1–11 (2019). 10.3390/molecules24162894 PMC6719981 31395831

[119] Schito, A. M. & Alfei, S. Antibacterial activity of non-cytotoxic, amino acid-modified polycationic dendrimers against *Pseudomonas aeruginosa* and other non-fermenting Gram-Negative bacteria. *Polymers* **12**, 1818 (2020). 32823557 10.3390/polym12081818 PMC7464783

[120] Pires, J. et al. In vitro activity of the novel antimicrobial peptide dendrimer g3kl against multidrug-resistant *Acinetobacter baumannii* and *Pseudomonas aeruginosa*. *Antimicrob. Agents Chemother.* **59**, 7915–7918 (2015). 26459893 10.1128/AAC.01853-15 PMC4649178

[121] Siriwardena, T. N. et al. Lipidated peptide dendrimers killing multidrug-resistant bacteria. *J. Am. Chem. Soc.* **140**, 423–432 (2018). 29206041 10.1021/jacs.7b11037

[122] Li, Q. Application of fragment-based drug discovery to versatile targets. *Front. Mol. Biosci.* **7**, 180 (2020). 32850968 10.3389/fmolb.2020.00180 PMC7419598

[123] Moreau, R. J. et al. Fragment-based drug discovery of inhibitors of phosphopantetheine adenylyltransferase from Gram-negative bacteria. *J. Med. Chem.* **61**, 3309–3324 (2018). 29498517 10.1021/acs.jmedchem.7b01691

[124] Mansbach, R. A. et al. Development of a fragment-based machine learning algorithm for designing hybrid drugs optimized for permeating Gram-negative bacteria. *arXiv*. 1907.13459 (2019).

[125] Mawal, Y., Critchley, I. A., Riccobene, T. A. & Talley, A. K. Ceftazidime-avibactam for the treatment of complicated urinary tract infections and complicated intra-abdominal infections. *Expert Rev. Clin. Pharmacol.* **8**, 691–707 (2015). 26420166 10.1586/17512433.2015.1090874

[126] Yaghoubi, S. et al. Tigecycline antibacterial activity, clinical effectiveness, and mechanisms and epidemiology of resistance: narrative review. *Eur. J. Clin. Microbiol. Infect. Dis.* **41**, 1003–1022 (2022). 33403565 10.1007/s10096-020-04121-1 PMC7785128

[127] Patel, T. S., Pogue, J. M., Mills, J. P. & Kaye, K. S. Meropenem-vaborbactam: a new weapon in the war against infections due to resistant Gram-negative bacteria. *Future Microbiol.* **13**, 971–983 (2018). 29692218 10.2217/fmb-2018-0054 PMC6161103

[128] Kashuba, A. D., Nafziger, A. N., Drusano, G. L. & Bertino, J. S. J. Optimizing aminoglycoside therapy for nosocomial pneumonia caused by Gram-negative bacteria. *Antimicrob. Agents Chemother.* **43**, 623–629 (1999). 10049277 10.1128/aac.43.3.623 PMC89170

[129] Sorbera, M., Chung, E., Ho, C. W. & Marzella, N. Ceftolozane/Tazobactam: a new option in the treatment of complicated Gram-negative infections. *J. Formulary Manag.* **39**, 825–832 (2014). PMC4264669 25516692

[130] Harris, P. N. A. et al. Effect of piperacillin-tazobactam vs meropenem on 30-day mortality for patients with *E. coli* or *Klebsiella pneumoniae* bloodstream infection and ceftriaxone resistance: a randomized clinical trial. *JAMA* **320**, 984–994 (2018). 30208454 10.1001/jama.2018.12163 PMC6143100

[131] Mansour, H., Ouweini, A. E. L., Chahine, E. B. & Karaoui, L. R. Imipenem/cilastatin/relebactam: a new carbapenem β-lactamase inhibitor combination. *Am. J. Health-Syst. Pharm.* **78**, 674–683 (2021). 33580649 10.1093/ajhp/zxab012

[132] Giacobbe, D. R. et al. Use of colistin in adult patients: a cross-sectional study. *J. Glob. Antimicrob. Resist.* **20**, 43–49 (2020). 31207379 10.1016/j.jgar.2019.06.009

[133] Greenberg, R. N., Reilly, P. M., Weinandt, W. J., Bollinger, M. & Kennedy, D. J. Cefoperazone-sulbactam combination in the treatment of urinary tract infections: efficacy, safety, and effects on coagulation. *Clin. Ther.* **10**, 52–56 (1987). 3329965

[134] Alosaimy, S., Abdul-Mutakabbir, J. C., Kebriaei, R., Jorgensen, S. C. J. & Rybak, M. J. Evaluation of eravacycline: a novel fluorocycline. *Pharmacotherapy* **40**, 221–238 (2020). 31944332 10.1002/phar.2366

[135] Wu, J. Y., Srinivas, P. & Pogue, J. M. Cefiderocol: a novel agent for the management of multidrug-resistant Gram-negative organisms. *Infect. Dis. Ther.* **9**, 17–40 (2020). 32072491 10.1007/s40121-020-00286-6 PMC7054475

[136] Goldstein, E. J. et al. Introduction of ertapenem into a hospital formulary: effect on antimicrobial usage and improved in vitro susceptibility of *Pseudomonas aeruginosa*. *Antimicrob. Agents Chemother.* **53**, 5122–5126 (2009). 19786596 10.1128/AAC.00064-09 PMC2786360

[137] Zhao, Y. et al. Cooperative membrane damage as a mechanism for pentamidine-antibiotic mutual sensitization. *ACS Chem. Biol.* **17**, 3178–3190 (2022). 36269311 10.1021/acschembio.2c00613

[138] Jones, R. N., Huynh, H. K. & Biedenbach, D. J. Activities of doripenem (S-4661) against drug-resistant clinical pathogens. *Antimicrob. Agents Chemother.* **48**, 3136–3140 (2004). 15273134 10.1128/AAC.48.8.3136-3140.2004 PMC478547

[139] Hilas, O., Ezzo, D. C. & Jodlowski, T. Z. Doripenem (doribax), a new carbapenem antibacterial agent. *P & T Peer-Rev. J. Formul. Manag.* **33**, 134–180 (2008). PMC2730083 19750153

[140] Perry, C. M. & Ibbotson, T. Biapenem. *Drugs* **62**, 2221–2234 (2002). 12381221 10.2165/00003495-200262150-00005

[141] Thakare, R., Singh, S., Dasgupta, A. & Chopra, S. Lascufloxacin hydrochloride to treat bacterial infection. *Drugs Today* **56**, 365–376 (2020). 10.1358/dot.2020.56.6.3137167 32525135

[142] Hosogaya, N. et al. Evaluation of efficacy and safety of lascufloxacin for nursing and healthcare associated pneumonia: single-arm, open-label clinical trial: a study protocol. *Medicine* **102**, e33092 (2023). 36827005 10.1097/MD.0000000000033092 PMC11309609

[143] Zhanel, G. G. et al. Lefamulin: a novel oral and intravenous pleuromutilin for the treatment of community-acquired bacterial pneumonia. *Drugs* **81**, 233–256 (2021). 33247830 10.1007/s40265-020-01443-4

[144] Clark, J. A. & Burgess, D. S. Plazomicin: a new aminoglycoside in the fight against antimicrobial resistance. *Ther. Adv. Infect. Dis.* **4**, 2049936120952604 (2020). 10.1177/2049936120952604 PMC7475792 32953108

[145] Sutcliffe, J. A., O’Brien, W., Fyfe, C. & Grossman, T. H. Antibacterial activity of eravacycline (TP-434), a novel fluorocycline, against hospital and community pathogens. *Antimicrob. Agents Chemother.* **57**, 5548–5558 (2013). 23979750 10.1128/AAC.01288-13 PMC3811277

[146] Basseatti, M., Ginocchio, F. & Mikulska, M. New treatment options against Gram-negative organisms. *Crit. Care* **15**, 215 (2011). 21457501 10.1186/cc9997 PMC3219411

[147] Butler, M. S. et al. Analysis of the clinical pipeline of treatments for drug-resistant bacterial infections: despite progress, more action is needed. *Antimicrob. Agents Chemother.* **66**, e0199121 (2022). 35007139 10.1128/aac.01991-21 PMC8923189

[148] Shi, S. et al. Synergistic effect of the novel β-lactamase inhibitor BLI-489 combined with imipenem or meropenem against diverse carbapenemase-producing carbapenem-resistant Enterobacterales. *J. Antimicrob. Chemother.* **77**, 1301–1305 (2022). 35165715 10.1093/jac/dkac037

[149] Richter, H. G. et al. Design, synthesis, and evaluation of 2 beta-alkenyl penam sulfone acids as inhibitors of beta-lactamases. *J. Med. Chem.* **39**, 3712–3722 (1996). 8809160 10.1021/jm9601967

[150] Kaur, K., Adediran, S. A., Lan, M. J. K. & Pratt, R. F. Inhibition of beta-lactamases by monocyclic acyl phosph(on)ates. *Biochemistry* **42**, 1529–1536 (2003). 12578365 10.1021/bi020602q

[151] Buynak, J. D., Ghadachanda, V. R., Vogeti, L., Zhang, H. & Chen, H. Synthesis and evaluation of 3-(carboxymethylidene)- and 3-(carboxymethyl)penicillinates as inhibitors of beta-lactamase. *J.Organ. Chem.* **70**, 4510–4513 (2005). 10.1021/jo050004s 15903334

[152] Livermore, D. M., Mushtaq, S. & Warner, M. Activity of BAL30376 (monobactam BAL19764 + BAL29880 + clavulanate) versus Gram-negative bacteria with characterized resistance mechanisms. *J. Antimicrob. Chemother.* **65**, 2382–2395 (2010). 20846937 10.1093/jac/dkq310

[153] Paukner, S., Hesse, L., Prezelj, A., Solmajer, T. & Urleb, U. In vitro activity of LK-157, a novel tricyclic carbapenem as broad-spectrum {beta}-lactamase inhibitor. *Antimicrob. Agents Chemother.* **53**, 505–511 (2009). 19075067 10.1128/AAC.00085-08 PMC2630636

[154] Simpson, I. N. et al. Synthesis and biological activity of AM-112 and related oxapenem analogues. *J. Antibiot.* **56**, 838–847 (2003). 10.7164/antibiotics.56.838 14700277

[155] *A*rixa pharmaceuticals announces acquisition by Pfizer’s hospital business _ business wire. (n.d.). https://www.businesswire.com/news/home/20201022005157/en/Arixa-Pharmaceuticals-Announces-Acquisition-by-Pfizer%E2%80%99s-Hospital-Business.

[156] Sader, H. S., Mendes, R. E., Duncan, L. R., Carvalhaes, C. G. & Castanheria, M. Antimicrobial activity of cefepime/zidebactam (WCK 5222), a β-lactam/β-lactam enhancer combination, against clinical isolates of Gram-negative bacteria collected worldwide (2018-19). *J. Antimicrob. Chemother.* **77**, 2642–2649 (2022). 35897129 10.1093/jac/dkac233

[157] Petropoulou, D., Siopi, M., Vourli, S. & Pournaras, S. Activity of sulbactam-durlobactam and comparators against a national collection of carbapenem-resistant *Acinetobacter baumannii* isolates from Greece. *Front. Cell. Infect. Microbiol.* **11**, 814530 (2021). 35127562 10.3389/fcimb.2021.814530 PMC8812809

[158] Hamrick, J. C. et al. VNRX-5133 (Taniborbactam), a broad-spectrum inhibitor of serine- and metallo-β-lactamases, restores activity of cefepime in Enterobacterales and *Pseudomonas aeruginosa*. *Antimicrob. Agents Chemother.* **64**, e01963–19 (2020). 31871094 10.1128/AAC.01963-19 PMC7038240

[159] Mallalieu, N. L. et al. Safety and pharmacokinetic characterization of nacubactam, a novel β-lactamase inhibitor, alone and in combination with meropenem, in healthy volunteers. *Antimicrob. Agents Chemother.* **64**, e02229–19 (2020). 32041717 10.1128/AAC.02229-19 PMC7179653

[160] O’Donnell, J. et al. Pharmacokinetic/pharmacodynamic determination and preclinical pharmacokinetics of the β-lactamase inhibitor ETX1317 and its orally available prodrug ETX0282. *ACS Infect. Dis.* **6**, 1378–1388 (2020). 32379415 10.1021/acsinfecdis.0c00019 PMC7297445

[161] Karlowsky, J. A., Hackel, M. A. & Sahm, D. F. In vitro activity of ceftibuten/VNRX-5236 against urinary tract infection isolates of antimicrobial-resistant Enterobacterales. *Antimicrob. Agents Chemother.* **66**, e0130421 (2022). 34662183 10.1128/AAC.01304-21 PMC8765315

[162] P1 single and multiple ascending dose (SAD_MAD) Study of IV QPX7728 alone and combined with QPX2014 in NHV. https://clinicaltrials.gov/ct2/show/NCT04380207.

[163] Livermore, D. M. et al. Activities of NXL104 combinations with ceftazidime and aztreonam against carbapenemase-producing Enterobacteriaceae. *Antimicrob. Agents Chemother.* **55**, 390–394 (2011). 21041502 10.1128/AAC.00756-10 PMC3019623

[164] Yamada, K. et al. In vivo efficacy of biapenem with ME1071, a novel metallo-β-lactamase (MBL) inhibitor, in a murine model mimicking ventilator-associated pneumonia caused by MBL-producing *Pseudomonas aeruginosa*. *Int. J. Antimicrob. Agents* **42**, 238–243 (2013). 23891525 10.1016/j.ijantimicag.2013.05.016

[165] Bruss, J. et al. Single- and multiple-ascending-dose study of the safety, tolerability, and pharmacokinetics of the polymyxin derivative SPR206. *Antimicrob. Agents Chemother.* **65**, e0073921 (2021). 34339267 10.1128/AAC.00739-21 PMC8448162

[166] Lepak, A. J., Wang, W. & Andes, D. R. Pharmacodynamic evaluation of MRX-8, a novel polymyxin, in the neutropenic mouse thigh and lung infection models against Gram-negative pathogens. *Antimicrob. Agents Chemother.* **64**, e01517–e01520 (2020). 32868332 10.1128/AAC.01517-20 PMC7577140

[167] Huband, M. D. et al. In vitro activity of KBP-7072, a novel third-generation tetracycline, against 531 recent geographically diverse and molecularly characterized *Acinetobacter baumannii* species complex isolates. *Antimicrob. Agents Chemother.* **64**, e02375–19 (2020). 32071042 10.1128/AAC.02375-19 PMC7179608

[168] Huband, M. D. et al. Activity of the novel aminomethylcycline KBP-7072 and comparators against 1,057 geographically diverse recent clinical isolates from the SENTRY surveillance program, 2019. *Antimicrob. Agents Chemother.* **66**, e0139721 (2022). 34633850 10.1128/AAC.01397-21 PMC8765295

[169] Hennessen, F. et al. Overcomes resistance mechanisms exerted on tetracyclines and natural chelocardin. *Antibiotics* **9**, 619 (2020). 32962088 10.3390/antibiotics9090619 PMC7559539

[170] Becker, K. et al. Efficacy of EBL-1003 (apramycin) against *Acinetobacter baumannii* lung infections in mice. *Clin. Microbiol. Infect.* **27**, 1315–1321 (2021). 33316399 10.1016/j.cmi.2020.12.004

[171] Richter, M. F. & Hergenrother, P. J. The challenge of converting Gram-positive-only compounds into broad-spectrum antibiotics. *Ann. N.Y. Acad. Sci.* **1435**, 18–38 (2019). 29446459 10.1111/nyas.13598 PMC6093809

[172] Acred, P., Brown, D. M., Turner, D. H. & Wilson, M. J. Pharmacology and chemotherapy of ampicillin-a new broad-spectrum penicillin. *Brit. J. Pharmacol. Chemother.* **18**, 356–369 (1962). 13859205 10.1111/j.1476-5381.1962.tb01416.x PMC1482127

[173] Savage, V. J. et al. Biological profiling of novel tricyclic inhibitors of bacterial DNA gyrase and topoisomerase IV. *J. Antimicrob. Chemother.* **71**, 1905–1913 (2016). 27032669 10.1093/jac/dkw061

[174] Richter, M. F. et al. Predictive compound accumulation rules yield a broad-spectrum antibiotic. *Nature* **545**, 299–304 (2017). 28489819 10.1038/nature22308 PMC5737020

[175] Parkinso, N. E. I. et al. Deoxynybomycins inhibit mutant DNA gyrase and rescue mice infected with fluoroquinolone-resistant bacteria. *Nat. Commun.* **6**, 6947 (2015). 25907309 10.1038/ncomms7947 PMC4421842

[176] Hiramatsu, K. et al. Curing bacteria of antibiotic resistance: reverse antibiotics, a novel class of antibiotics in nature. *Int. J. Antimicrob. Agents* **39**, 478–485 (2012). 22534508 10.1016/j.ijantimicag.2012.02.007

[177] Parker, E. N. et al. Implementation of permeation rules leads to a FabI inhibitor with activity against Gram-negative pathogens. *Nature Microbiol.* **5**, 67–75 (2020). 31740764 10.1038/s41564-019-0604-5 PMC6953607

[178] Howe, J. A. et al. Selective small-molecule inhibition of an RNA structural element. *Nature* **526**, 672–67 (2015). 26416753 10.1038/nature15542

[179] Tari, L. W. et al. Tricyclic GyrB/ParE (TriBE) inhibitors: a new class of broad-spectrum dual-targeting antibacterial agents. *PloS One* **8**, e84409 (2013). 24386374 10.1371/journal.pone.0084409 PMC3873466

[180] Onishi, H. R. et al. Antibacterial agents that inhibit lipid A biosynthesis. *Science* **274**, 980–982 (1996). 8875939 10.1126/science.274.5289.980

[181] A study to assess the safety, tolerability, and pharmacokinetics of ACHN-975 in healthy volunteers. https://clinicaltrials.gov/ct2/show/NCT01597947?term=NCT01597947&draw=2&rank=1.

[182] A multiple dose study to assess the safety, tolerability, and pharmacokinetics of ACHN-975 in healthy volunteers https://clinicaltrials.gov/ct2/show/NCT01870245?term=NCT01870245&draw=1&rank=1.

[183] Birck, M. R., Holler, T. P. & Woodard, R. W. Identification of a slow tight-binding inhibitor of 3-deoxy-D-manno-octulosonic acid 8-phosphate synthase. *J. Am. Chem. Soc.* **122**, 9334–9335 (2000).

[184] Novartis licenses three novel anti-infective programs to Boston Pharmaceuticals https://www.novartis.com/news/media-releases/novartis-licenses-three-novel-anti-infective-programs-boston-pharmaceuticals (2018).

[185] Lehman, K. M. & Grabowicz, M. Countering Gram-negative antibiotic resistance: recent progress in disrupting the outer membrane with novel therapeutics. *Antibiotics* **8**, 163 (2019). 31554212 10.3390/antibiotics8040163 PMC6963605

[186] Xiao, Y., Gerth, K., Müller, R. & Wall, D. Myxobacterium-produced antibiotic TA (myxovirescin) inhibits type II signal peptidase. *Antimicrob. Agents Chemother.* **56**, 2014–2021 (2012). 22232277 10.1128/AAC.06148-11 PMC3318312

[187] Vogeley, L. et al. Structural basis of lipoprotein signal peptidase II action and inhibition by the antibiotic globomycin. *Science* **351**, 876–880 (2016). 26912896 10.1126/science.aad3747

[188] Ho, H. et al. Structural basis for dual-mode inhibition of the ABC transporter MsbA. *Nature* **557**, 196–201 (2018). 29720648 10.1038/s41586-018-0083-5

[189] Mandler, M. D. et al. Novobiocin enhances polymyxin activity by stimulating lipopolysaccharide transport. *J. Am. Chem. Soc.* **140**, 6749–6753 (2018). 29746111 10.1021/jacs.8b02283 PMC5990483

[190] Martin-Loeches, I., Dale, G. E. & Torres, A. Murepavadin: a new antibiotic class in the pipeline. *Expert Rev. Anti-Infect. Ther.* **16**, 259–268 (2018). 29451043 10.1080/14787210.2018.1441024

[191] Srinivas, N. et al. Peptidomimetic antibiotics target outer-membrane biogenesis in *Pseudomonas aeruginosa*. *Science* **327**, 1010–1013 (2010). 20167788 10.1126/science.1182749

[192] Vetterli, S. U. et al. Thanatin targets the intermembrane protein complex required for lipopolysaccharide transport in *Escherichia coli*. *Sci. Adv.* **4**, eaau2634 (2018). 30443594 10.1126/sciadv.aau2634 PMC6235536

[193] Sherman, D. J., Okuda, S., Denny, W. A. & Kahne, D. Validation of inhibitors of an ABC transporter required to transport lipopolysaccharide to the cell surface in *Escherichia coli*. *Bioorgan. Med. Chem.* **21**, 4846–4851 (2013). 10.1016/j.bmc.2013.04.020 PMC3821166 23665139

[194] Nickerson, N. N. et al. A Novel Inhibitor of the LolCDE ABC Transporter Essential for Lipoprotein Trafficking in Gram-Negative Bacteria. *Antimicrob. Agents Chemother.* **62**, e02151–17 (2018). 29339384 10.1128/AAC.02151-17 PMC5913989

[195] Pathania, R. et al. Chemical genomics in *Escherichia coli* identifies an inhibitor of bacterial lipoprotein targeting. *Nat. Chem. Biol.* **5**, 849–856 (2009). 19783991 10.1038/nchembio.221

[196] Barker, C. A. et al. Degradation of MAC13243 and studies of the interaction of resulting thiourea compounds with the lipoprotein targeting chaperone LolA. *Bioorgan. Med. Chem. Lett.* **23**, 2426–2431 (2013). 10.1016/j.bmcl.2013.02.005 23473681

[197] Buss, J. A. et al. Pathway-directed screen for inhibitors of the bacterial cell elongation machinery. *Antimicrob. Agents Chemother.* **63**, e01530–18 (2019). 30323039 10.1128/AAC.01530-18 PMC6325226

[198] Hart, E. M. et al. A small-molecule inhibitor of BamA impervious to efflux and the outer membrane permeability barrier. *Proc. Natl Acad. Sci. USA* **116**, 21748–21757 (2019). 31591200 10.1073/pnas.1912345116 PMC6815139

[199] Hagan, C. L., Wzorek, J. S. & Kahne, D. Inhibition of the β-barrel assembly machine by a peptide that binds BamD. *Proc. Natl Acad. Sci. USA* **112**, 2011–2016 (2015). 25646443 10.1073/pnas.1415955112 PMC4343090

[200] Sader, H. S. et al. Antimicrobial activity of POL7306 tested against clinical isolates of Gram-negative bacteria collected worldwide. *J. Antimicrob. Chemother.* **75**, 1518–1524 (2020). 32087024 10.1093/jac/dkaa020

[201] Tomasek, D. et al. Structure of a nascent membrane protein as it folds on the BAM complex. *Nature* **583**, 473–478 (2020). 32528179 10.1038/s41586-020-2370-1 PMC7367713

